# Conditioning Intensity, Pre-Transplant Flow Cytometric Measurable Residual Disease, and Outcome in Adults with Acute Myeloid Leukemia Undergoing Allogeneic Hematopoietic Cell Transplantation

**DOI:** 10.3390/cancers12092339

**Published:** 2020-08-19

**Authors:** Linde M. Morsink, Brenda M. Sandmaier, Megan Othus, Raffaele Palmieri, Noa Granot, Evandro D. Bezerra, Brent L. Wood, Marco Mielcarek, Gary Schoch, Chris Davis, Mary E. D. Flowers, H. Joachim Deeg, Frederick R. Appelbaum, Rainer Storb, Roland B. Walter

**Affiliations:** 1 Clinical Research Division, Fred Hutchinson Cancer Research Center, Seattle, WA 98109, USA; lmorsin2@fredhutch.org (L.M.M.); bsandmai@fredhutch.org (B.M.S.); rpalmier@fredhutch.org (R.P.); ngranot@fredhutch.org (N.G.); mmielcar@fredhutch.org (M.M.); gschoch@fredhutch.org (G.S.); cdavis@fredhutch.org (C.D.); mflowers@fredhutch.org (M.E.D.F.); jdeeg@fredhutch.org (H.J.D.); fappelba@fredhutch.org (F.R.A.); rstorb@fredhutch.org (R.S.); 2 Department of Hematology, University Medical Center Groningen, 9700 RB Groningen, The Netherlands; 3 Division of Medical Oncology, Department of Medicine, University of Washington, Seattle, WA 98195, USA; 4 Public Health Science Division, Fred Hutchinson Cancer Research Center, Seattle, WA 98109, USA; mothus@fredhutch.org; 5 Department of Medicine, Residency Program, University of Washington, Seattle, WA 98195, USA; bezerra.evandro@mayo.edu; 6 Department of Laboratory Medicine and Pathology, University of Washington, Seattle, WA 98195, USA; woodbl@uw.edu; 7 Division of Hematology, Department of Medicine, University of Washington, Seattle, WA 98195, USA; 8 Department of Epidemiology, University of Washington, Seattle, WA 98195, USA

**Keywords:** acute myeloid leukemia (AML), adults, allogeneic, conditioning, hematopoietic cell transplantation, intensity, measurable (minimal) residual disease, multiparameter flow cytometry

## Abstract

How conditioning intensity is related to outcomes of AML patients undergoing allografting in morphologic remission is an area of great ongoing interest. We studied 743 patients in morphologic remission and known pre-transplant measurable residual disease (MRD) status determined by multiparameter flow cytometry (MFC) who received a first allograft after myeloablative, reduced intensity, or nonmyeloablative conditioning (MAC, RIC, and NMA). Overall, relapse-free survival (RFS) and overall survival (OS) were longer after MAC than RIC or NMA conditioning, whereas relapse risks were not different. Among MRD^pos^ patients, 3-year estimates of relapse risks and survival were similar across conditioning intensities. In contrast, among MRD^neg^ patients, 3-year RFS and OS were longer for MAC (69% and 71%) than RIC (47% and 55%) and NMA conditioning (47% and 52%). Three-year relapse risks were lowest after MAC (18%) and highest after NMA conditioning (30%). Our data indicate an interaction between conditioning intensity, MFC-based pre-transplant MRD status, and outcome, with benefit of intensive conditioning primarily for patients transplanted in MRD^neg^ remission. Differing from recent findings from other studies that indicated MAC is primarily beneficial for some or all patients with MRD^pos^ pre-HCT status, our data suggest MAC should still be considered for MRD^neg^ AML patients if tolerated.

## 1. Introduction

Many adults with acute myeloid leukemia (AML) in morphologic remission are treated with allogeneic hematopoietic cell transplantation (HCT) [1,2]. While an important cornerstone of curative-intent therapy, however, AML relapse has remained a problem following allografting [1]. This is particularly true for patients with evidence of measurable (‘minimal’) residual disease (MRD), as detected by multiparameter flow cytometry (MFC) or molecular methods, at the time of HCT [3,4,5,6]. Consequently, identifying strategies to improve these post-transplant outcomes is a major focus of current research efforts.

Several retrospective studies of patients non-randomly assigned to receive higher- or lower-intensity conditioning regimens suggested lower relapse rates with myeloablative conditioning (MAC) compared to reduced-intensity conditioning (RIC) or nonmyeloablative (NMA) conditioning [7,8,9,10,11]. Congruent with these findings, data from the randomized phase 3 BMT CTN 0901 trial showed, for adults age 18–65 years with AML transplanted in morphologic remission, MAC was associated with lower relapse rates and longer survival compared to RIC [12]. In a recent subsequent post-hoc analysis of a subset of 190 AML patients (>70% older than age 50) for whom pre-transplant peripheral blood specimens were archived, Hourigan et al. used ultra-deep, error-corrected sequencing of 13 commonly mutated genes in AML as an approach to test for mutation-defined MRD before HCT [13]. Results showed that there was a statistically significantly lower incidence of relapse as well as longer relapse-free survival (RFS) and overall survival (OS) with MAC in the 66% of patients entering transplantation with genomic evidence of residual AML (or, more specifically, the 41% of patients with mutations present in genes other than *DNMT3A*, *TET2*, and *ASXL1*). On the other hand, in the patients without genomic MRD, MAC was associated with only a statistically non-significant improvement in relapse incidence and, related to higher non-relapse mortality (NRM), similar OS compared to RIC [13].

The findings from the BMT CTN 0901 trial are partially consistent with those from a retrospective analysis of registry data from the Acute Leukemia Working Party of the European Society of Blood and Marrow Transplantation (EBMT) that included 2292 adults with AML undergoing allogeneic HCT in first morphologic remission, many of whom (>45%) given in vivo T-cell depleted allografts [14]. In this cohort, there was a reduced incidence of relapse and better RFS with MAC relative to RIC in MRD^pos^ but not MRD^neg^ patients, with MRD status assessed by individual participating centers using molecular and/or MFC assays. However, in this EBMT analysis, this benefit was only seen in individuals younger than age 50, whereas there was no benefit for MAC in patients older than age 50, regardless of MRD status at the time of HCT [14]. A single institution study of 203 patients with AML in morphologic remission undergoing either umbilical cord blood or sibling donor HCT also suggested that conditioning intensity impacts the post-HCT outcomes of patients with pre-transplant MRD by showing the MFC-based MRD status was not associated with relapse or decreased OS after MAC whereas, in the subset of patients receiving RIC HCT, those with MRD experienced significantly higher rates of relapse and shorter RFS and OS compared to those without MRD [15].

In apparent disagreement with these data, we found no statistically significant difference for the association between MRD, as quantified by MFC, and relapse risk with conditioning intensity for 86 patients undergoing nonmyeloablative (NMA) conditioning and 155 patients undergoing MAC before allogeneic HCT for AML in first remission in a retrospective analysis reported previously [16]. Since that analysis was limited by the relatively small sample size, we here used information from nearly 750 consecutive adults undergoing allogeneic HCT with bone marrow or G-CSF mobilized peripheral blood stem cells while in morphologic remission at our institution between 2006 and 2019 to re-examine the relationship between conditioning intensity, pre-HCT MRD status, and post-transplant outcomes.

## 2. Results

### 2.1. Patient and Transplant Characteristics

For our study, we initially identified 763 patients. Of these, 10 did not agree to their data being used for research purposes and 10 did not undergo MRD testing at our institution during the pre-HCT work-up, leaving 743 patients who received MAC (*n* = 441), RIC (*n* = 130) or NMA conditioning (*n* = 172) HCT for analysis. Table 1 provides a summary of the characteristics of the study population, donors, and transplants.

Not unexpectedly, there were several statistically significant differences between the three conditioning intensity groups, including age at diagnosis and at time of HCT (younger in MAC patients; *p* < 0.001), white blood cell (WBC) count at diagnosis (higher in MAC patients; *p* < 0.001), the proportion of individuals with secondary AML (lower in MAC patients; *p* = 0.0015), the duration of CR before HCT (longer in NMA HCT patients; *p* = 0.0061), HCT comorbidity index (lower in MAC patients; *p* < 0.001), and the proportion of patients with recovered peripheral blood counts before HCT (higher in MAC patients; *p* = 0.035). There were also significant differences in the proportion of unrelated donor HCT and degrees of HLA matching (*p* = 0.003 and *p* < 0.001, respectively), source of stem cells (*p* < 0.001), and type of GVHD prophylaxis (*p* < 0.001). However, the proportions of patients testing positive for MRD before HCT were similar across these groups (*p* = 0.22).

### 2.2. Association between MRD Status, Conditioning Intensity, and Post-HCT Outcome

**Figure 1:**
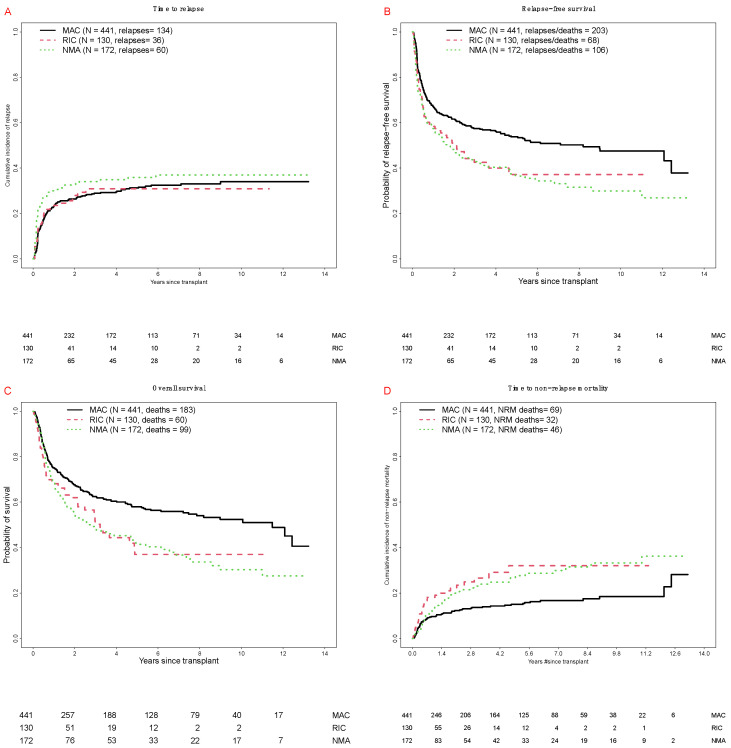
Post-transplant outcomes for 743 adults with AML undergoing allogeneic HCT while in first or second morphologic remission, stratified by conditioning intensity. (**A**) Cumulative risk of relapse. (**B**) Relapse-free survival. (**C**) Overall survival. (**D**) Cumulative risk of non-relapse mortality. Outcome estimates are shown separately for MAC patients (*n* = 441), RIC patients (*n* = 130), and NMA HCT patients (*n* = 172), respectively.

There were 342 deaths, 230 relapses, and 147 NRM events contributing to the probability estimates for relapse, OS, RFS, and NRM. In our cohort, median follow-up after HCT among survivors was 56 (3–159) months: 67 (3–159) months for MAC, 25 (4–136) months for RIC, and 50 (3–158) months for NMA HCT patients, respectively. Overall, as summarized in Figure 1 and Table 2, there were no statistically significant differences in the cumulative incidence of relapse at 3 years when patients were stratified according to conditioning intensity. However, MAC patients had higher 3-year estimates of RFS and OS than patients who received RIC or NMA conditioning, whereas 100-day NRM rates were similar for the three different conditioning intensities.

**Figure 2:**
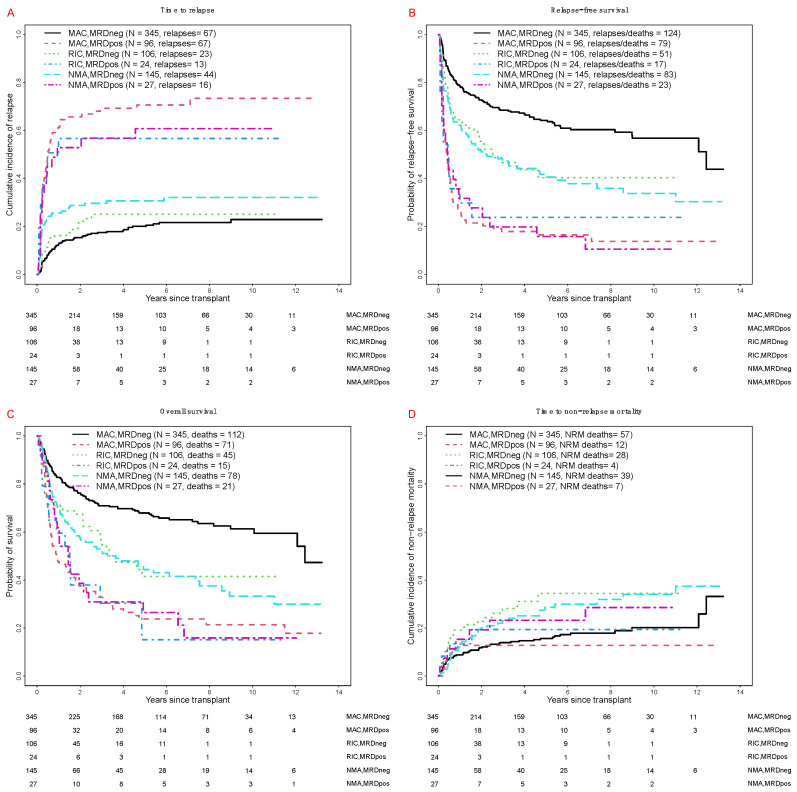
Post-transplant outcomes for 743 adults with AML undergoing allogeneic HCT while in first or second morphologic remission, stratified by conditioning intensity and pre-transplant MRD status. (**A**) Cumulative risk of relapse. (**B**) Relapse-free survival, (**C**) Overall survival. (**D**) Cumulative risk of non-relapse mortality. Outcome estimates are shown separately for MAC patients in MRD^neg^ remission (*n* = 345) or MRD^pos^ remission (*n* = 96), RIC patients in MRD^neg^ remission (*n* = 106) or MRD^pos^ remission (*n* = 24), and NMA HCT patients in MRD^neg^ remission (*n* = 145) or MRD^pos^ remission (*n* = 27), respectively.

Consistent with previous reports from our institution [16,17,18,19,20,21,22,23,24], patients testing MRD^pos^ at the time of HCT had a substantially higher risk of relapse and shorter RFS and OS than MRD^neg^ patients (Table 2). Among MRD^pos^ patients, 3-year estimates of relapse risk, RFS and OS were similar across the 3 conditioning intensities (Table 2 and Figure 2). On the other hand, there were significant differences in these outcomes across conditioning intensities among MRD^neg^ patients. Specifically, the 3-year cumulative incidence of relapse was lower after MAC than NMA, with the relapse risk after RIC being in between the relapse risk estimates for MAC and NMA (Table 2 and Figure 2A). 3-year RFS estimates were higher among MRD^neg^ MAC patients than RIC and NMA conditioning patients; Figure 2B), as were the 3-year OS estimates (Figure 2C).

### 2.3. Relationship between Pre-HCT MRD Status and Conditioning Regimen

To study the relationship between pre-transplant MRD status, intensity of the conditioning regimen, and post-HCT outcomes in more detail, we built uni- and multivariable regression models for the endpoints of relapse, failure for RFS, and overall mortality. As summarized in Table 3 for the entire study cohort, the unadjusted hazard ratios of RIC vs. MAC and NMA vs. MAC were 0.98 (0.68–1.41; *p* = 0.91) and 1.27 (0.93–1.73; *p* = 0.13) for relapse, 1.45 (1.10–1.91; *p* = 0.009) and 1.57 (1.24–1.99; *p* < 0.001) for failure of RFS, and 1.50 (1.12–2.01; *p* = 0.007) as well as 1.56 (1.22–1.99; *p* < 0.001) for overall mortality, respectively. Similar to our previous studies, being MRD^pos^ was associated with higher risk of relapse, failure for RFS, and overall mortality in univariate models (all *p* < 0.001). We also found statistically significant associations between the outcomes of interest (relapse, RFS, and/or OS) and several other covariates, including remission status (second vs. first remission), cytogenetic risk (adverse vs. favorable/intermediate), age, pre-HCT karyotype for patients with cytogenetically abnormal AML (not normalized vs. normalized), and blood counts before HCT (recovered vs. not recovered) (Table 3).

After adjustment for various covariates as summarized in Table 4, hazard ratios for RIC vs. MAC and NMA vs. MAC were 1.46 (0.89–2.40; *p* = 0.14) and 2.40 (1.51–3.73; *p* < 0.001) for relapse, 1.81 (1.27–2.58; *p* = 0.0011) and 2.10 (1.52–2.92; *p* < 0.001) for RFS failure, and 1.72 (1.18–2.51; *p* = 0.0048) as well as 1.82 (1.30–2.56; *p* < 0.001) for overall mortality. As before, being MRD^pos^ was associated with higher risk of relapse, failure of RFS, and overall mortality (all *p* < 0.001]). Importantly, the observation that relapse risks and survival outcomes appeared similar among MRD^pos^ patients across the three conditioning intensities but differed for MRD^neg^ patients indicated an interaction between conditioning intensity and pre-HCT MRD status. This notion was supported by the results from an interaction term between the intensity of the conditioning regimen and pre-transplant MRD status that was included in the multivariable models, with statistically significant findings for the interaction between NMA conditioning and MRD status for relapse (*p* = 0.011), RFS (*p* = 0.0089), and OS (*p* = 0.012).

### 2.4. Relationship between MRD Status and Pre-HCT Conditioning Regimen in Different Subsets of Patients

Finally, we examined the outcomes of patients treated with different pre-HCT conditioning regimens in distinct patient subsets of the study cohort. Such subsets of interest included the 570 patients transplanted in first remission and the 626 patients who underwent allografting from fully HLA-matched donors. Additionally, we performed a subset analysis restricting our patients to those who received similar conditioning regimens as those used in the BMT CTN 0901 trial and, as applied in the BMT CTN 0901 trial, had an HCT-CI score of ≤4 and received HLA-identical/matched or 1-allele/antigen mismatched allografts [12]. These subset analyses included 215 patients who received MAC with high-dose TBI (with/without cyclophosphamide or fludarabine; *n* = 28) or cyclophosphamide in combination with 4 days of busulfan or fludarabine (*n* = 187). They also included 20 RIC patients who received conditioning with either fludarabine/melphalan (*n* = 11) or 2 days of busulfan and fludarabine (*n* = 9). As the small sample size would have precluded meaningful analyses, we also included, in the RIC group, patients conditioned with fludarabine/melphalan/low-dose TBI (*n* = 17) and patients receiving clofarabine/low-dose TBI (*n* = 28), for a total of 65 RIC patients. The results of these subset analyses are summarized in Tables S1–S3 and Figures S1 and S2 (for the subset of first remission patients), Tables S4–S6 and Figures S3 and S4 (for the subset of patients who received fully HLA-matched allografts), and Tables S7–S9 and Figures S5 and S6 (for the subset of MAC and RIC patients conditioned with regimens similar to those used in the BMT CTN 0901 trial). The findings in these subset analyses were similar to those obtained in the overall analysis, with better survival estimates following MAC than RIC or NMA primarily as a result of lower rates of deaths without prior relapse with RIC/NMA compared to MAC. Again, in all of these analyses, there was no apparent benefit of higher intensity conditioning in the subset of MRD^pos^ patients.

## 3. Discussion

One area of current interest in the care of adults with AML in morphologic remission planned to undergo allogeneic HCT relates to the intensity of the conditioning therapy that provides optimal outcomes and, particularly, the question whether or not patients with and those without pre-HCT MRD should be approached differently. Findings from several studies, including a recent analysis of a subset of patients participating in the randomized BMT CTN 0901 trial, suggested benefit of MAC primarily in some or all patients presenting with MRD at the time of allografting [13,14,15]. In contrast to these data, in the present large retrospective analysis in which we determined the pre-HCT MRD status via MFC, 3-year estimates of relapse risk, RFS, and OS were similar across the three conditioning intensities examined among MRD^pos^ patients. Also similar were the 100-day NRM rates in the three conditioning intensity cohorts. Yet, among MRD^neg^ patients, RFS and OS were longer after MAC than RIC or NMA conditioning after multivariable adjustments because of a lower relapse incidence and a lower incidence of deaths without prior recorded relapse at later times after MAC as compared to RIC/NMA conditioning.

Several factors could account for the differences between our results and those observed in the previous studies by others. Unlike the BMT CTN 0901 study (but similar to the EBMT and University of Minnesota cohorts), assignment to different conditioning intensity was done in a non-random fashion in our cohort, leading to different patient and disease characteristics among recipients of MAC, RIC, and NMA HCT. In fact, the lower incidence of deaths without prior relapse at later times noted in our study after MAC as compared to RIC/NMA conditioning was likely a reflection of differences in characteristics of patients eligible for MAC HCT as compared to RIC and NMA HCT that we could only partially account for in our analyses—an important distinction from the prospective randomized BMT CTN 0901 trial where patients had to be eligible for either arm of the randomization [12]. There are also notable differences in the specifics of conditioning regimens and post-transplant therapies (e.g., T-cell depletion of allografts, GVHD prophylaxis) between individual studies. It is also possible specifics of the assay used to determine MRD status and, with that, the subsets of patients deemed to have/not have MRD at the time of HCT, could contribute to inter-study differences. In what way the source of material used to test for MRD (peripheral blood vs. bone marrow) could influence results is unknown.

At first glance, our observation suggesting MAC may be particularly beneficial for patients without MRD at the time of HCT may be counterintuitive. However, fundamentally, testing MRD^pos^ at the time of allogeneic HCT by MFC is a marker of measurably suboptimal response to non-transplant therapy (most commonly, conventional chemotherapy). Our current findings suggest, therefore, that in these patients with relatively chemotherapy-resistant disease, currently used high-intensity conditioning regimens—which, by themselves, are often chemotherapy-based—are unable to overcome this resistance. However, an earlier prospective, randomized trial comparing a currently used regimen of cyclophosphamide and 12 Gy fractionated TBI to an ultra-high-intensity regimen of cyclophosphamide and 15.75 Gy fractionated TBI in patients with AML in morphologic CR (MRD was not determined) showed a significantly reduced rate of relapse among patients given the higher TBI dose [25,26]. Unfortunately, this therapeutic gain was offset by a significantly higher rate of NRM from regimen-related toxicities. While comparison with that study must consider the time gap of almost three decades, results suggest that, if conditioning intensity could be increased without increasing toxicity, relapse rates might be reduced, and survival improved, particularly among MRD^pos^ AML patients. On the other hand, our data are consistent with the notion that in patients with more chemotherapy-sensitive disease, as indicated by achievement of a remission without MFC evidence of MRD, intensifying conditioning is effective in reducing disease burdens further.

Since 2006, multiparameter flow cytometry-based MRD testing on bone marrow specimens is routinely performed as part of the pre-HCT work-up in our institution in a largely unchanged fashion. As a strength of our study, this allowed us to include essentially all adults with AML undergoing allogeneic HCT in the analysis. Results from MRD testing were available to transplant teams. However, while MRD was increasingly recognized as a relevant prognostic marker over the last 15 years, the MRD status typically played no major role in the selection of the type of preparative regimen. That is because no MRD-directed transplant protocols were available, and patients with AML were routinely assigned to myeloablative conditioning unless significant comorbidities were present; only a small number of patients were enrolled in trials comparing different intensities of conditioning regimens. There are important limitations of our study to acknowledge. These include its retrospective nature and the fact that transplant protocol assignments were done in a non-randomized fashion. Other limitations are the relatively short follow-up time for patients transplanted most recently and the lack of data on molecular profiles of the patients’ leukemias. The latter is because most of patients were referred to our institution for transplantation after they received induction and consolidation chemotherapy elsewhere. Particularly in the earlier years of the 2006–2019 time period, many of these patients did not undergo detailed (or any) molecular testing at the time of diagnosis. Because of the relatively small number of patients <65 years of age receiving allografts after RIC or NMA conditioning, we were also unable to perform subset analyses of the younger individuals in our study cohort. For example, among patients <50 years of age, only 18 and 13 patients underwent RIC and NMA HCT, respectively. Among patients <65 years of age, 76 and 77 patients underwent RIC and NMA HCT, respectively, but the number of patients with positive pre-HCT MRD test was too small to enable reliable comparisons (6 with RIC and 15 with NMA HCT).

## 4. Materials and Methods

### 4.1. Study Cohort

Adults ≥18 years of age with AML (based on 2016 WHO criteria [27]) were included in our analysis provided they were in first or second morphologic remission (i.e., <5% blasts in the bone marrow, no circulating blasts, and no evidence of extramedullary leukemia) and underwent a first allogeneic HCT with peripheral blood or bone marrow as a stem cell source between 4/2006 (the time a refined ten-color MFC-based MRD assay was introduced and routinely employed in all HCT patients) and 12/2019. Secondary AML was defined as disease following an antecedent hematologic disorder (e.g., myelodysplastic syndrome or myeloproliferative neoplasm) or treatment with systemic chemotherapy and/or radiotherapy for a different disorder [16,20]. Patients and related or unrelated donors were selected by high-resolution HLA-typing. The HCT-specific comorbidity index (HCT-CI) was calculated as previously described [28]. Treatment responses were categorized as proposed by the European LeukemiaNet [2]. Information on post-transplant outcomes was captured via the Long-Term Follow-Up Program through medical records from our outpatient clinic and local clinics that provided primary care for patients in addition to records obtained on patients on research studies. In previous publications, we have reported partial results from 667 of the 743 patients included in this study cohort [16,17,18,19,20,21,22,23,24]. Patients were treated either on Institutional Review Board-approved research protocols (all registered with ClinicalTrials.gov) or on standard treatment protocols. All gave consent for treatment in accordance with the Declaration of Helsinki. Follow-up was as of 27 February 2020. This retrospective analysis was approved by the Institutional Review Board at Fred Hutchinson Cancer Research Center (protocol #2562).

### 4.2. Types and Intensity of Conditioning Regimens

High-dose fractionated total body irradiation (TBI; ≥12 Gy) with or without cyclophosphamide (CY) or fludarabine (FLU), high-dose TBI/thiotepa/FLU, busulfan (4 days) with CY or FLU, treosulfan/FLU with or without low-dose TBI, or any regimen containing a radiolabeled antibody were considered MAC regimens. NMA regimens included 2–3 Gy TBI with or without fludarabine. All others were considered RIC regimens.

### 4.3. Classification of Disease Risk at Diagnosis and Cytogenetic Analysis at the Time of HCT

Cytogenetic risk at the time of AML diagnosis was assigned using the refined MRC/NCRI criteria [29] and was based on local cytogenetic data. Three hundred and twenty seven of the 743 patients included in our final data set had a normal karyotype. These included 273 patients with ≥20 normal metaphases examined (*n* = 273) and 54 patients with <20 metaphases examined. Following the approach described by Breems and colleagues [30], we considered all of these patients to have cytogenetically normal AML. As part of the pre-HCT work-up, bone marrow specimens were obtained and examined for cytogenetic abnormalities. Standard G-banding techniques were used, and samples were karyotyped according to the International System for Human Cytogenetic Nomenclature [31].

### 4.4. Detection of MRD by Multiparameter Flow Cytometry

As a routine clinical test, bone marrow aspirates were obtained in all patients before conditioning therapy was started for MRD analysis via 10-color flow cytometry [16,17,18,20,21,32]. As we reported previously [17,32], the MRD assay included monoclonal antibodies recognizing CD4, CD5, CD7, CD13, CD14, CD15, CD16, CD19, CD33, CD34, CD38, CD45, CD56, CD64, CD71, CD117, CD123, and HLA-DR. The panel consisted of three tubes as follows: (1) CD13-PC7, CD15-FITC, CD19-PE-CF594, CD33-PE, CD34-APC, CD38-A594, CD45-APC-H7, CD71-APC-A700, CD117-PC5, and HLA-DR-PB; (2) CD4-ECD, CD13-PC7, CD14-Cy5.5, CD16-APC-A700, CD34-APC, CD38-A594, CD45-APC-H7, CD64-FITC, CD123-PE, and HLA-DR-PB; and (3) CD5-PC5, CD7-PE, CD33-PC7, CD34-APC, CD38-A594, CD45-APC-H7, and CD56-A488. All antibodies were obtained from Beckman Coulter (Indianapolis, IN) or Becton Dickinson (BD Biosciences, San Jose, CA). Up to 1 million events per tube were acquired on a custom-built LSR II flow cytometer (BD Biosciences). Data compensation and analysis was performed with noncommercial software (WoodList; developed by B.L.W.). MRD was identified by visual inspection as a cell population showing deviation (typically seen in more than one antigen) from the normal patterns of antigen expression found on specific cell lineages at specific stages of maturation as compared with either normal or regenerating marrow based on the tested antibody panel [32]. In a large majority of cases, this assay detects MRD to a level of 0.1%. In progressively smaller subsets of patients, this assay also detects MRD below that level. An identified abnormal cell population was quantified as a percentage of the total CD45^+^ white cell events. We considered *any* measurable level of MRD to be positive, consistent with the approach we have taken previously [16,17,18,20,21].

### 4.5. Statistical Analysis

The Kaplan–Meier method was used to estimate unadjusted probabilities of RFS and OS. Post-transplant relapse was defined as emergence of any level of disease for MRD^neg^ patients and persistent/worsening disease burden or treated disease for MRD^pos^ patients. Probabilities of NRM and relapse were summarized using cumulative incidence estimates. NRM was defined as death without prior relapse and was considered a competing risk for relapse, while relapse was a competing risk for NRM. Associations with RFS and OS were assessed using Cox regression, while proportional subdistribution hazard models that account for competing risks assessed associations with relapse and NRM. Besides conditioning intensity (MAC vs. RIC vs. NMA), covariates evaluated were: pre-HCT MRD (yes vs. no), first vs. second remission at time of HCT, cytogenetic risk group at time of AML diagnosis (favorable/intermediate vs. adverse), type of AML at diagnosis (secondary vs. de novo), karyotype at time of HCT (normalized vs. not normalized for patients presenting with abnormal karyotypes), peripheral blood counts at the time of HCT (recovered [i.e., absolute neutrophil count >1000/µL and platelet count >100,000/µL] vs. not recovered), age at time of HCT, HCT-CI (0–1 vs. 2–3 vs. ≥4), and WBC count at the time of diagnosis. We compared categorical patient characteristics with Fisher’s exact tests. Wilcoxon rank sum tests were used to compare quantitative characteristics. Two-sided *p*-values are reported throughout; no adjustment for multiple comparisons was made in any analysis. STATA 16.1 (StataCorp LP, College Station, TX, USA) and R (R Foundation for Statistical Computing, Vienna, Austria; http://www.r-project.org) were used to perform all statistical analyses.

## 5. Conclusions

Acknowledging the limitations of our study, our analyses show an interaction of conditioning intensity, MFC-based pre-HCT MRD status, and outcome for adults with AML undergoing allografting while in morphologic remission, with benefit of intensive conditioning (MAC) over lower-intensity conditioning (RIC/NMA) primarily for patients transplanted in remission without MFC evidence of MRD. Differing from recent findings from other analyses, our data indicate that high-intensity conditioning should still be considered for patients with MRD^neg^ pre-HCT status if not precluded by concerns of transplant-related toxicity.

## Figures and Tables

**Table 1:** Pre-transplantation demographic and clinical characteristics of the entire study cohort, stratified by conditioning intensity.

Parameter	MAC (*n* = 441)	RIC (*n* = 130)	NMA (*n* = 172)	All Patients (*n* = 743)	*p*-Value
**Median age at diagnosis (range), years**	49 (18–71)	62 (20–74)	65 (19–77)	55 (18–77)	<0.001
**Median age at HCT (range), years**	50 (18–73)	63 (23–75)	66 (20–80)	56 (18–80)	<0.001
**Male gender, *n* (%)**	230 (52)	67 (52)	105 (61)	402 (54)	0.11
**Median WBC count at diagnosis (range), ×10^3^/µL**	9 (0–297)	5 (0–348)	4 (1–295)	7 (0–348)	<0.001
**Cytogenetics, *n* (%)**					0.90
Favorable	32 (7)	6 (5)	8 (5)	46 (6)	
Intermediate	276 (63)	84 (65)	111 (65)	471 (63)	
Adverse	112 (25)	35 (27)	45 (26)	192 (26)	
Missing	21 (5)	5 (4)	8 (5)	34 (5)	
**Remission status, *n* (%)**					0.14
First remission	334 (76)	95 (73)	141 (82)	570 (77)	
Second remission	107 (24)	35 (27)	31 (18)	173 (23)	
**Pre-HCT MRD status, *n* (%)**					0.22
MRD^neg^	345 (78)	106 (82)	145 (84)	596 (80)	
MRD^pos^	96 (22)	24 (18)	27 (16)	147 (20)	
Median % abnormal blasts (range)	0.49 (0.007–19.4)	0.66 (0.007–5)	0.21 (0.01–2.7)	0.5 (0.007–19.4)	0.13
**Secondary AML, *n* (%)**	102 (23)	46 (35)	62 (36)	210 (28)	0.0015
**Median remission duration before HCT (range), days**	95 (7–485)	83 (11–455)	108 (16–788)	96 (7–788)	0.0061
Recovered peripheral blood counts before HCT *, *n* (%)	328 (74)	88 (68)	111 (65)	527 (71)	0.035
Recovered ANC before HCT *, *n* (%)	412 (93)	116 (89)	159 (92)	687 (92)	0.28
Recovered platelet count before HCT *, *n* (%)	330 (75)	90 (69)	113 (66)	533 (72)	0.061
**Routine cytogenetics before HCT, *n* (%)**					0.44
Normalized karyotype	174 (39)	46 (35)	62 (36)	282 (38)	
Abnormal karyotype	78 (18)	27 (21)	22 (13)	127 (17)	
Non-informative karyotype **	173 (39)	53 (41)	80 (47)	306 (41)	
Missing	16 (4)	4 (3)	8 (5)	28 (4)	
**HCT Comorbidity Index, *n* (%)**					<0.001
0–1	114 (26)	14 (11)	36 (21)	164 (22)	
2–3	164 (37)	52 (40)	52 (30)	268 (36)	
≥4	110 (25)	52 (40)	77 (45)	239 (32)	
Missing	53 (12)	12 (9)	7 (4)	72 (10)	
**Unrelated donor, *n* (%)**	277 (63)	88 (68)	132 (77)	497 (67)	0.003
**HLA matching, *n* (%)**					<0.001
Related donors					
*HLA-identical ^#^*	155 (35)	25 (19)	40 (23)	220 (30)	
*HLA-haploidentical*	9 (2)	17 (13)	0 (0)	26 (3)	
Unrelated donors					
*10/10 HLA-matched*	230 (52)	78 (60)	104 (60)	412 (55)	
*9/10 HLA-matched ^##^*	47 (11)	10 (8)	28 (16)	85 (11)	
**Patient/donor CMV status**					0.80
Neg/neg	114 (26)	25 (20)	45 (26)	184 (25)	
Neg/pos	51 (12)	19 (15)	20 (12)	90 (12)	
Pos/neg	140 (32)	42 (34)	57 (33)	239 (33)	
Pos/pos	126 (29)	39 (31)	49 (29)	214 (29)	
**Conditioning regimen, *n* (%)**					<0.001
MAC, with high-dose TBI (≥12 Gy)	62 (14)	---	---	62 (8)	
MAC, without high-dose TBI	379 (86)	---	---	379 (51)	
RIC	---	130 (100)	---	130 (17)	
NMA	---	---	172 (100)	172 (23)	
**Source of stem cells, *n* (%)**					<0.001
PBSC	374 (85)	121 (93)	172 (100)	667 (90)	
BM	67 (15)	9 (7)	0 (0)	76 (10)	
**GVHD prophylaxis, *n* (%)**					<0.001
CNI + MMF ± sirolimus	60 (14)	81 (62)	166 (97)	307 (41)	
CNI + MTX ± other	310 (70)	23 (18)	0 (0)	333 (45)	
PTCy	58 (13)	26 (20)	5 (3)	89 (12)	
Other	13 (3)	0 (0)	1 (1)	14 (2)	

* ANC ≥ 1000/µL and platelets ≥ 100,000/µL. ** Normal cytogenetics in patient with cytogenetically normal AML or missing cytogenetics at diagnosis. ^#^ 6 of the siblings had an antigen mismatch resulting from a crossover event. ^##^ 63 had antigen level and 32 had allele level HLA-mismatch; 3 with DR mismatch had an additional DQ mismatch and 1 with HLA-A antigen mismatch also had HLA-B allele mismatch. ANC: absolute neutrophil count; BM: bone marrow; CNI: calcineurin inhibitor; HCT: hematopoietic cell transplantation; MAC: myeloablative conditioning; MMF: mycophenolate mofetil; MTX: methotrexate; NMA: nonmyeloablative; PBSC: peripheral blood stem cells; PTCy: post transplantation cyclophosphamide; RIC: reduced-intensity conditioning; TBI: total body irradiation; WBC: white blood cell. *p*-values for quantitative covariates were calculated using Wilcoxon rank sum tests, *p*-values for categorical variable were calculated using Fisher’s exact test.

**Table 2:** Post-transplant outcomes of the entire study cohort, stratified by intensity of the conditioning regimen and pre-transplant MRD status.

Cohort	CI of Relapse at 3 Years	RFS at 3 Years	OS at 3 Years	CI of NRM at 100 Days	CI of NRM at 3 Years
**All patients**					
All (*n* = 743)	30% (27–34)	52% (48–56)	57% (54–61)	5% (3–6)	18% (15–21)
MRDneg (*n* = 596)	22% (18–25)	60% (56–64)	64% (60–68)	5% (3–6)	18% (15–22)
MRDpos (*n* = 147)	65% (57–74)	19% (13–27)	32% (25–41)	5% (1–8)	16% (10–22)
**MAC HCT**					
All (*n* = 441)	29% (25–33)	57% (53–62)	62% (58–67)	5% (3–6)	14% (10–17)
MRDneg (*n* = 345)	18% (13–22)	69% (64–74)	71% (66–76)	5% (3–7)	10% (7–14)
MRDpos (*n* = 96)	69% (60–79)	18% (12–28)	33% (24–44)	3% (0–7)	14% (7–21)
**RIC HCT**					
All (*n* = 130)	31% (22–40)	43% (34–54)	50% (41–62)	7% (3–11)	27% (18–35)
MRDneg (*n* = 106)	25% (16–35)	47% (37–59)	55% (44–67)	7% (3–11)	28% (18–38)
MRDpos (*n* = 24)	57% (35–79)	24% (11–53)	30% (15–62)	8% (0–20)	19% (1–38)
**NMA HCT**					
All (*n* = 172)	34% (27–41)	43% (36–51)	48% (41–57)	4% (1–6)	23% (16–30)
MRDneg (*n* = 145)	30% (22–37)	47% (39–57)	52% (44–61)	3% (0–5)	23% (16–30)
MRDpos (*n* = 27)	57% (37–77)	20% (9–43)	31% (17–55)	7% (0–18)	23% (6–40)

CI: cumulative incidence; HCT: hematopoietic cell transplantation; MAC: myeloablative conditioning; MRD: measurable residual disease; NMA: nonmyeloablative; NRM: non-relapse mortality; OS: overall survival; RFS: relapse-free survival; RIC: reduced-intensity conditioning. Shown are point estimates (95% confidence intervals).

**Table 3:** Univariate regression models built for the entire study cohort.

Covariate	Relapse	Failure for RFS	Overall Mortality
**Conditioning regimen**			
MAC (*n* = 441)	1 (Reference)	1 (Reference)	1 (Reference)
RIC (*n* = 130)	0.98 (0.68–1.41), *p* = 0.91	1.45 (1.10–1.91), *p* = 0.009	1.50 (1.12–2.01), *p* = 0.007
NMA (*n* = 172)	1.27 (0.93–1.73), *p* = 0.13	1.57 (1.24–1.99), *p* <0.001	1.56 (1.22–1.99), *p* < 0.001
**Pre-HCT MRD status**			
MRD^neg^ (*n* = 596)	1 (Reference)	1 (Reference)	1 (Reference)
MRD^pos^ (*n* = 147)	4.26 (3.27–5.56), *p* < 0.001	3.18 (2.55–3.96), *p* < 0.001	2.47 (1.96–3.10), *p* < 0.001
**Remission status**			
First remission (*n* = 570)	1 (Reference)	1 (Reference)	1 (Reference)
Second remission (*n* = 173)	1.47 (1.10–1.96), *p* = 0.009	1.47 (1.17–1.84), *p* = 0.001	1.50 (1.18–1.89), *p* < 0.001
**Cytogenetic risk**			
Favorable/intermediate (*n* = 517)	1 (Reference)	1 (Reference)	1 (Reference)
Adverse (*n* = 192)	2.02 (1.55–2.65), *p* < 0.001	1.34 (1.07–1.68), *p* = 0.01	1.20 (0.95–1.53), *p* = 0.13
**Age at HCT (per 10 years)**	1.00 (0.99–1.01), *p* = 0.82	1.01 (1.01–1.02), *p* = 0.001	1.02 (1.01–1.02), *p* < 0.001
**Total WBC count at diagnosis (per 10,000/µL)**	1.00 (1.00–1.00), *p* = 0.67	1.00 (1.00–1.00), *p* = 0.072	1.00 (1.00–1.00), *p* = 0.046
**HCT Comorbidity Index**			
0–1 (*n* = 164)	1 (Reference)	1 (Reference)	1 (Reference)
2–3 (*n* = 268)	0.97 (0.70–1.36), *p* = 0.86	1.06 (0.81–1.39), *p* = 0.66	1.09 (0.82–1.45), *p* = 0.54
≥4 (*n* = 239)	0.96 (0.61–1.68), *p* = 0.96	1.22(0.93–1.60), *p* = 0.16	1.32 (0.99–1.76), *p* = 0.06
**Type of AML**			
De novo (*n* = 533)	1 (Reference)	1 (Reference)	1 (Reference)
Secondary (*n* = 210)	0.95 (0.71–1.27), *p* = 0.74	1.07 (0.85–1.33), *p* = 0.57	1.10 (0.87–1.38), *p* = 0.44
**Pre-HCT karyotype**			
Normalized (*n* = 281)	1 (Reference)	1 (Reference)	1 (Reference)
Not normalized (*n* = 127)	2.00 (1.43–2.81), *p* < 0.001	2.10 (1.59–2.76), *p* < 0.001	1.99 (1.49–2.67), *p* < 0.001
Pre-HCT blood counts *			
Recovered (*n* = 527)	1 (Reference)	1 (Reference)	1 (Reference)
Not recovered (*n* = 216)	0.95 (0.71–1.27), *p* = 0.74	1.33 (1.07–1.65), *p* = 0.009	1.49 (1.19–1.86), *p* < 0.001
**Donor type**			
Related (*n* = 246)	1 (Reference)	1 (Reference)	1 (Reference)
Unrelated (*n* = 497)	0.94 (0.72–1.23), *p* = 0.65	1.14 (0.92–1.42), *p* = 0.22	1.20 (0.96–1.51), *p* = 0.11
**HLA matching**			
Matched/identical (*n* = 630)	1 (Reference)	1 (Reference)	1 (Reference)
9/10 matched (*n* = 85)	1.13 (0.79–1.62), *p* = 0.51	1.69 (1.29–2.21), *p* < 0.001	1.80 (1.36–2.39), *p* < 0.001
Haploidentical (*n* = 26)	1.54 (0.86–2.77), *p* = 0.15	1.73 (1.06–2.83), *p* = 0.028	1.84 (1.09–3.10), *p* = 0.021

* Recovered: ANC ≥ 1000/µL and platelets ≥100,000/µL; not recovered: ANC < 1000/µL and/or platelets < 100,000/µL. ANC: absolute neutrophil count; HCT: hematopoietic cell transplantation; MAC: myeloablative conditioning; MRD: measurable residual disease; NMA: nonmyeloablative; RFS: relapse free survival; RIC: reduced-intensity conditioning; WBC: white blood cell. *p*-values were calculated from Cox (RFS, OS) and Fine and Gray (relapse) regression models.

**Table 4:** Multivariable regression models for relapse, failure for RFS, and overall mortality in the entire study cohort.

Covariate	Relapse	Failure for RFS	Overall Mortality
**Conditioning regimen**			
MAC	1 (Reference)	1 (Reference)	1 (Reference)
RIC	1.46 (0.89–2.40), *p* = 0.14	1.81 (1.27–2.58), *p* = 0.0011	1.72 (1.18–2.51), *p* = 0.0048
NMA	2.40 (1.55–3.73), *p* < 0.001	2.10 (1.52–2.92), *p* < 0.001	1.82 (1.30–2.56), *p* < 0.0048
**Pre-HCT MRD status**			
MRD^neg^	1 (Reference)	1 (Reference)	1 (Reference)
MRD^pos^	5.67 (4.03–7.99), *p* < 0.001	4.02 (2.98–5.44), *p* < 0.001	3.00 (2.19–4.12), *p* < 0.001
**Remission status**			
First remission	1 (Reference)	1 (Reference)	1 (Reference)
Second remission	1.62 (1.15–2.26), *p* = 0.005	1.45 (1.13–1.87), *p* = 0.004	1.35 (1.04–1.76), *p* = 0.026
**Cytogenetic risk**			
Favorable/intermediate	1 (Reference)	1 (Reference)	1 (Reference)
Adverse	1.87 (1.30–2.69), *p* < 0.001	1.30 (0.98–1.73), *p* = 0.065	1.24 (0.93–1.67), *p* = 0.15
**Age at HCT (per 10 years)**	0.94 (0.84–1.04), *p* = 0.21	1.02 (0.93–1.11), *p* = 0.70	1.05 (0.96–1.16), *p* = 0.29
**Total WBC count at diagnosis (per 10,000/µL)**	1.01 (0.99–1.04), *p* = 0.23	1.03 (1.01–1.04), *p* = 0.0036	1.02 (1.01–1.04), *p* = 0.011
**HCT Comorbidity Index**			
Low	1 (Reference)	1 (Reference)	1 (Reference)
Intermediate	0.95 (0.68–1.32), *p* = 0.76	1.03 (0.78–1.35), *p* = 0.85	1.04 (0.78–1.39), *p* = 0.79
High	0.92 (0.64–1.32), *p* = 0.63	1.09 (0.82–1.45), *p* = 0.56	1.20 (0.89–1.62), *p* = 0.23
**Type of AML**			
De novo	1 (Reference)	1 (Reference)	1 (Reference)
Secondary	0.70 (0.51–0.96), *p* = 0.028	0.79 (0.62–1.02), *p* = 0.071	0.87 (0.67–1.12), *p* = 0.27
**Pre-HCT karyotype**			
Normalized	1 (Reference)	1 (Reference)	1 (Reference)
Not normalized	1.53 (1.07–2.18), *p* = 0.019	1.64 (1.21–2.21), *p* = 0.0013	1.50 (1.09–2.07), *p* = 0.012
Pre-HCT blood counts *			
Recovered	1 (Reference)	1 (Reference)	1 (Reference)
Not recovered	0.80 (0.57–1.13), *p* = 0.20	1.04 (0.82–1.10), *p* = 0.76	1.25 (0.98–1.59), *p* = 0.076
**Interaction RIC-MRD^pos^**	0.65 (0.28–1.52), *p* = 0.32	0.62 (0.32–1.17), *p* = 0.14	0.52 (0.26–1.05), *p* = 0.067
**Interaction NMA-MRD^pos^**	0.42 (0.21–0.82), *p* = 0.011	0.47 (0.27–0.83), *p* = 0.0089	0.47 (0.26–0.85), *p* = 0.012

* Recovered: ANC ≥ 1000/µL and platelets ≥ 100,000/µL; not recovered: ANC < 1000/µL and/or platelets < 100,000/µL. ANC: absolute neutrophil count; HCT: hematopoietic cell transplantation; MAC: myeloablative conditioning; MRD: measurable residual disease; NMA: nonmyeloablative; RFS: relapse free survival; RIC: reduced-intensity conditioning; WBC: white blood cell. *p*-values were calculated from Cox (RFS, OS) and Fine and Gray (relapse) regression models.

## Supplementary Materials

The following are available online at https://www.mdpi.com/2072-6694/12/9/2339/s1, Figure S1: Post-transplant outcome for 570 adults with AML undergoing allogeneic HCT while in first morphologic remission, stratified by conditioning intensity. Figure S2: Post-transplant outcome for 570 adults with AML undergoing allogeneic HCT while in first morphologic remission, stratified by conditioning intensity and pre-transplant MRD status. Figure S3: Post-transplant outcome for 626 adults with AML undergoing HCT with an HLA-identical/matched allograft, stratified by conditioning intensity. Figure S4: Post-transplant outcome for 626 adults with AML undergoing HCT with an HLA-identical/matched allograft, stratified by conditioning intensity and pre-transplant MRD status. Figure S5: Post-transplant outcome for 280 adults with AML undergoing allogeneic HCT while in first or second morphologic remission after receiving MAC or RIC regimens similar to those used in the BMT CTN 0901 trial, stratified by conditioning intensity. Figure S6: Post-transplant outcomes for 280 adults with AML undergoing allogeneic HCT while in first or second morphologic remission after receiving MAC or RIC regimens similar to those used in the BMT CTN 0901 trial, stratified by conditioning intensity and pre-transplant MRD status. Table S1: Pre-transplantation demographic and clinical characteristics for the subset of patients transplanted in first remission, stratified by conditioning intensity. Table S2: Univariate regression models for the subset of patients transplanted in first remission. Table S3: Multivariable regression models for the subset of patients transplanted in first remission. Table S4: Demographic and clinical characteristics of the subset of patients who underwent fully HLA-matched related- or unrelated-donor HCT, stratified by conditioning intensity. Table S5: Univariate regression models for the subset of patients who underwent fully HLA-matched related- or unrelated-donor HCT. Table S6: Multivariable regression models for the subset of patients who underwent fully HLA-matched related- or unrelated-donor HCT. Table S7: Pre-transplantation demographic and clinical characteristics for the subset of patients who received MAC and RIC regimens similar to those used in the BMT CTN 0901 trial, stratified by conditioning intensity. Table S8: Univariate regression models for the subset of patients who received MAC and RIC regimens similar to those used in the BMT CTN 0901 trial. Table S9: Multivariable regression models for the subset of patients who received MAC and RIC regimens similar to those used in the BMT CTN 0901 trial.

## References

[1] Cornelissen J.J., Gratwohl A., Schlenk R.F., Sierra J., Bornhäuser M., Juliusson G., Råcil Z., Rowe J.M., Russell N., Mohty M. (2012). The European LeukemiaNet AML Working Party consensus statement on allogeneic HSCT for patients with AML in remission: An integrated-risk adapted approach. Nat. Rev. Clin. Oncol..

[2] Döhner H., Estey E., Grimwade D., Amadori S., Appelbaum F.R., Buchner T., Dombret H., Ebert B.L., Fenaux P., Larson R.A. (2017). Diagnosis and management of AML in adults: 2017 ELN recommendations from an international expert panel. Blood.

[3] Hourigan C.S., Gale R.P., Gormley N.J., Ossenkoppele G.J., Walter R.B. (2017). Measurable residual disease testing in acute myeloid leukaemia. Leukemia.

[4] Schuurhuis G.J., Heuser M., Freeman S., Béné M.C., Buccisano F., Cloos J., Grimwade D., Haferlach T., Hills R.K., Hourigan C.S. (2018). Minimal/measurable residual disease in AML: A consensus document from the European LeukemiaNet MRD Working Party. Blood.

[5] Buckley S.A., Wood B.L., Othus M., Hourigan C.S., Ustun C., Linden M.A., DeFor T.E., Malagola M., Anthias C., Valkova V. (2017). Minimal residual disease prior to allogeneic hematopoietic cell transplantation in acute myeloid leukemia: A meta-analysis. Haematologica.

[6] Thol F., Gabdoulline R., Liebich A., Klement P., Schiller J., Kandziora C., Hambach L., Stadler M., Koenecke C., Flintrop M. (2018). Measurable residual disease monitoring by NGS before allogeneic hematopoietic cell transplantation in AML. Blood.

[7] Aoudjhane M., Labopin M., Gorin N.C., Shimoni A., Ruutu T., Kolb H.J., Frassoni F., Boiron J.M., Yin J.L., Finke J. (2005). Comparative outcome of reduced intensity and myeloablative conditioning regimen in HLA identical sibling allogeneic haematopoietic stem cell transplantation for patients older than 50 years of age with acute myeloblastic leukaemia: A retrospective survey from the Acute Leukemia Working Party (ALWP) of the European group for Blood and Marrow Transplantation (EBMT). Leukemia.

[8] Shimoni A., Hardan I., Shem-Tov N., Yeshurun M., Yerushalmi R., Avigdor A., Ben-Bassat I., Nagler A. (2006). Allogeneic hematopoietic stem-cell transplantation in AML and MDS using myeloablative versus reduced-intensity conditioning: The role of dose intensity. Leukemia.

[9] Alyea E.P., Kim H.T., Ho V., Cutler C., DeAngelo D.J., Stone R., Ritz J., Antin J.H., Soiffer R.J. (2006). Impact of conditioning regimen intensity on outcome of allogeneic hematopoietic cell transplantation for advanced acute myelogenous leukemia and myelodysplastic syndrome. Biol. Blood Marrow Transpl..

[10] Ringdén O., Labopin M., Ehninger G., Niederwieser D., Olsson R., Basara N., Finke J., Schwerdtfeger R., Eder M., Bunjes D. (2009). Reduced intensity conditioning compared with myeloablative conditioning using unrelated donor transplants in patients with acute myeloid leukemia. J. Clin. Oncol..

[11] Luger S.M., Ringdén O., Zhang M.J., Pérez W.S., Bishop M.R., Bornhauser M., Bredeson C.N., Cairo M.S., Copelan E.A., Gale R.P. (2012). Similar outcomes using myeloablative vs reduced-intensity allogeneic transplant preparative regimens for AML or MDS. Bone Marrow Transpl..

[12] Scott B.L., Pasquini M.C., Logan B.R., Wu J., Devine S.M., Porter D.L., Maziarz R.T., Warlick E.D., Fernandez H.F., Alyea E.P. (2017). Myeloablative versus reduced-intensity hematopoietic cell transplantation for acute myeloid leukemia and myelodysplastic syndromes. J. Clin. Oncol..

[13] Hourigan C.S., Dillon L.W., Gui G., Logan B.R., Fei M., Ghannam J., Li Y., Licon A., Alyea E.P., Bashey A. (2020). Impact of conditioning intensity of allogeneic transplantation for acute myeloid leukemia with genomic evidence of residual disease. J. Clin. Oncol..

[14] Gilleece M.H., Labopin M., Yakoub-Agha I., Volin L., Socié G., Ljungman P., Huynh A., Deconinck E., Wu D., Bourhis J.H. (2018). Measurable residual disease, conditioning regimen intensity, and age predict outcome of allogeneic hematopoietic cell transplantation for acute myeloid leukemia in first remission: A registry analysis of 2292 patients by the Acute Leukemia Working Party European Society of Blood and Marrow Transplantation. Am. J. Hematol..

[15] Ustun C., Courville E.L., DeFor T., Dolan M., Randall N., Yohe S., Bejanyan N., Warlick E., Brunstein C., Weisdorf D.J. (2016). Myeloablative, but not reduced-intensity, conditioning overcomes the negative effect of flow-cytometric evidence of leukemia in acute myeloid leukemia. Biol. Blood Marrow Transpl..

[16] Walter R.B., Gyurkocza B., Storer B.E., Godwin C.D., Pagel J.M., Buckley S.A., Sorror M.L., Wood B.L., Storb R., Appelbaum F.R. (2015). Comparison of minimal residual disease as outcome predictor for AML patients in first complete remission undergoing myeloablative or nonmyeloablative allogeneic hematopoietic cell transplantation. Leukemia.

[17] Walter R.B., Gooley T.A., Wood B.L., Milano F., Fang M., Sorror M.L., Estey E.H., Salter A.I., Lansverk E., Chien J.W. (2011). Impact of pretransplantation minimal residual disease, as detected by multiparametric flow cytometry, on outcome of myeloablative hematopoietic cell transplantation for acute myeloid leukemia. J. Clin. Oncol..

[18] Walter R.B., Buckley S.A., Pagel J.M., Wood B.L., Storer B.E., Sandmaier B.M., Fang M., Gyurkocza B., Delaney C., Radich J.P. (2013). Significance of minimal residual disease before myeloablative allogeneic hematopoietic cell transplantation for AML in first and second complete remission. Blood.

[19] Walter R.B., Sandmaier B.M., Storer B.E., Godwin C.D., Buckley S.A., Pagel J.M., Sorror M.L., Deeg H.J., Storb R., Appelbaum F.R. (2015). Number of courses of induction therapy independently predicts outcome after allogeneic transplantation for acute myeloid leukemia in first morphological remission. Biol. Blood Marrow Transpl..

[20] Araki D., Wood B.L., Othus M., Radich J.P., Halpern A.B., Zhou Y., Mielcarek M., Estey E.H., Appelbaum F.R., Walter R.B. (2016). Allogeneic hematopoietic cell transplantation for acute myeloid leukemia: Is it time to move toward a minimal residual disease-based definition of complete remission. J. Clin. Oncol..

[21] Zhou Y., Othus M., Araki D., Wood B.L., Radich J.P., Halpern A.B., Mielcarek M., Estey E.H., Appelbaum F.R., Walter R.B. (2016). Pre- and post-transplant quantification of measurable (‘minimal’) residual disease via multiparameter flow cytometry in adult acute myeloid leukemia. Leukemia.

[22] Hoffmann A.P., Besch A.L., Othus M., Morsink L.M., Wood B.L., Mielcarek M., Estey E.H., Appelbaum F.R., Walter R.B. (2020). Early achievement of measurable residual disease (MRD)-negative complete remission as predictor of outcome after myeloablative allogeneic hematopoietic cell transplantation in acute myeloid leukemia. Bone Marrow Transpl..

[23] Morsink L.M., Othus M., Bezerra E.D., Wood B.L., Fang M., Sandmaier B.M., Mielcarek M., Schoch G., Storb R., Deeg H.J. (2020). Impact of pre-transplant measurable residual disease on outcome of allogeneic hematopoietic cell transplantation in adult monosomal karyotype AML. Leukemia.

[24] Morsink L.M., Bezerra E.D., Othus M., Wood B.L., Fang M., Sandmaier B.M., Mielcarek M.B., Deeg H.J., Schoch G., Appelbaum F.R. (2020). Comparative analysis of total body irradiation (TBI)-based and non-TBI-based myeloablative conditioning for acute myeloid leukemia in remission with or without measurable residual disease. Leukemia.

[25] Clift R.A., Buckner C.D., Appelbaum F.R., Bearman S.I., Petersen F.B., Fisher L.D., Anasetti C., Beatty P., Bensinger W.I., Doney K. (1990). Allogeneic marrow transplantation in patients with acute myeloid leukemia in first remission: A randomized trial of two irradiation regimens. Blood.

[26] Clift R.A., Buckner C.D., Appelbaum F.R., Sullivan K.M., Storb R., Thomas E.D. (1998). Long-term follow-up of a randomized trial of two irradiation regimens for patients receiving allogeneic marrow transplants during first remission of acute myeloid leukemia. Blood.

[27] Arber D.A., Orazi A., Hasserjian R., Thiele J., Borowitz M.J., Le Beau M.M., Bloomfield C.D., Cazzola M., Vardiman J.W. (2016). The 2016 revision to the World Health Organization classification of myeloid neoplasms and acute leukemia. Blood.

[28] Sorror M.L., Maris M.B., Storb R., Baron F., Sandmaier B.M., Maloney D.G., Storer B. (2005). Hematopoietic cell transplantation (HCT)-specific comorbidity index: A new tool for risk assessment before allogeneic HCT. Blood.

[29] Grimwade D., Hills R.K., Moorman A.V., Walker H., Chatters S., Goldstone A.H., Wheatley K., Harrison C.J., Burnett A.K. (2010). Refinement of cytogenetic classification in acute myeloid leukemia: Determination of prognostic significance of rare recurring chromosomal abnormalities among 5876 younger adult patients treated in the United Kingdom Medical Research Council trials. Blood.

[30] Breems D.A., Van Putten W.L.J., De Greef G.E., Van Zelderen-Bhola S.L., Gerssen-Schoorl K.B.J., Mellink C.H.M., Nieuwint A., Jotterand M., Hagemeijer A., Beverloo H.B. (2008). Monosomal karyotype in acute myeloid leukemia: A better indicator of poor prognosis than a complex karyotype. J. Clin. Oncol..

[31] Simons A., Shaffer L.G., Hastings R.J. (2013). Cytogenetic nomenclature: Changes in the ISCN 2013 compared to the 2009 edition. Cytogenet. Genome Res..

[32] Wood B.L. (2020). Acute myeloid leukemia minimal residual disease detection: The difference from normal approach. Curr. Protoc. Cytom..

