# Supplementary material for: Conditioning Intensity, Pre-Transplant Flow Cytometric Measurable Residual Disease, and Outcome in Adults with Acute Myeloid Leukemia Undergoing Allogeneic Hematopoietic Cell Transplantation
Source: Cancers (Basel). 2020 Aug 19;12(9):2339. doi: 10.3390/cancers12092339 (PMC7565021; doi:10.3390/cancers12092339)
Supplement: Supplementary file 1 [file cancers-12-02339-s001.pdf]

SUPPLEMENTARY TABLE 1. Pre-transplantation demographic and clinical characteristics for the subset of patients transplanted in first remission, stratified by conditioning intensity

	MAC (n=344)	RIC (n=95)	NMA (n=141)	All patients (n=570)	*P*-value
**Median age at diagnosis (range), years**	50 (18-71)	64 (28-74)	65 (19-77)	56 (18-77)	<0.001
**Median age at HCT (range), years**	50 (18-72)	64 (29-75)	66 (20-78)	56 (18-78)	<0.001
**Male gender, n (%)**	175 (52)	55 (58)	89 (63)	319 (56)	0.08
**Median WBC at diagnosis (range), x10^3/µL**	8 (0-280)	3 (0-317)	3 (1-295)	5 (0-317)	0.002
**Cytogenetics, n (%)**					0.82
Favorable	12 (4)	1 (1)	7 (5)	20 (4)	
Intermediate	207 (62)	60 (63)	86 (61)	353 (62)	
Adverse	100 (30)	31 (33)	42 (30)	173 (30)	
Missing	15 (4)	3 (3)	6 (4)	24 (4)	
**Pre-HCT MRD status, n (%)**					0.71
MRD^{neg}	269 (81)	77 (81)	118 (84)	464 (81)	
MRD^{pos}	65 (19)	18 (19)	23 (16)	106 (19)	
Median % abnormal blasts (range)	0.4 (0.007-19.4)	0.6 (0.007-4.8)	0.4 (0.02-2.7)	0.4 (0.007-19.4)	0.52
**Secondary AML, n (%)**	93 (28)	41 (43)	58 (41)	192 (34)	0.004
**Median CR duration before HCT (range), days**	110 (14-485)	92 (21-455)	114 (18-788)	110 (14-788)	0.11
**Recovered peripheral blood counts before HCT*, n (%)**	262 (78)	67 (71)	96 (68)	425 (75)	0.03
**Recovered ANC before HCT*, n (%)**	317 (95)	89 (94)	130 (92)	536 (94)	0.47
**Recovered platelet count before HCT*, n (%)**	264 (79)	68 (72)	98 (70)	430 (75)	0.05
**Routine cytogenetics before HCT, n (%)**					0.60
Normalized karyotype	149 (45)	36 (38)	57 (40)	242 (42)	
Abnormal karyotype	54 (16)	19 (20)	19 (13)	92 (16)	
Non-informative karyotype**	123 (37)	38 (40)	59 (42)	220 (39)	
Missing	8 (2)	2 (2)	6 (4)	16 (3)	
**HCT Comorbidity Index, n (%)**					<0.001
0-1	88 (26)	13 (14)	29 (21)	130 (23)	
2-3	124 (37)	36 (38)	44 (31)	204 (36)	
≥4	79 (24)	38 (40)	63 (45)	180 (32)	
Missing	43 (13)	8 (8)	5 (4)	56 (10)	
**Unrelated donor, n (%)**	213 (64)	68 (72)	107 (76)	388 (68)	0.03
**Patient / donor CMV status**					0.77
Neg / neg	85 (26)	20 (22)	37 (26)	142 (25)	
Neg / pos	40 (12)	17 (19)	16 (11)	73 (13)	

Pos / neg	102 (31)	29 (32)	46 (33)	177 (32)	
Pos / pos	100 (31)	25 (27)	41 (29)	166 (30)	
**Conditioning regimen, n (%)**					<0.001
MAC					
Containing high-dose TBI (≥12 Gy)	43 (13)	---	---	43 (8)	
Not containing high-dose TBI	291 (87)	---)	---	291 (51)	
RIC	---	95 (100)	---	95 (17)	
NMA	---	---	141 (100)	141 (25)	
**Source of stem cells, n (%)**					<0.001
PBSC	284 (85)	90 (95)	141 (100)	515 (90)	
BM	50 (15)	5 (5)	0 (0)	55 (10)	
**GVHD prophylaxis, n (%)**					<0.001
CNI + MMF ± sirolimus	38 (11)	61 (64)	135 (96)	234 (41)	
CNI + MTX ± other	245 (73)	15 (16)	0 (0)	260 (46)	
PTCy	41 (12)	19 (20)	5 (4)	65 (11)	
Other	10 (3)	0 (0)	1 (1)	11 (2)	

*ANC ≥1,000/µL and platelets ≥100,000/µL; **normal cytogenetics in patient with cytogenetically normal AML or missing cytogenetics at diagnosis. Abbreviations: ANC, absolute neutrophil count; BM, bone marrow; CNI, calcineurin inhibitor; HCT, hematopoietic cell transplantation; MAC, myeloablative conditioning; MMF, mycophenolate mofetil; MTX, methotrexate; NMA, nonmyeloablative; PBSC, peripheral blood stem cells; PTCy, post transplantation cyclophosphamide; RIC, reduced intensity conditioning; TBI, total body irradiation; WBC, total white blood cell count.

SUPPLEMENTARY TABLE 2. Univariate regression models for the subset of patients transplanted in first remission

	Relapse	Failure for RFS	Overall mortality
**Conditioning regimen**			
MAC (n=334)	1 (Reference)	1 (Reference)	1 (Reference)
RIC (n=95)	0.88 (0.56-1.39), *P*=0.59	1.33 (0.95-1.86), *P*=0.10	1.41 (0.99-2.02), *P*=0.06
NMA (n=141)	1.15 (0.80-1.66), *P*=0.44	1.52 (1.16-1.99), *P*=0.002	1.57 (1.18-2.09), *P*=0.002
**Pre-HCT MRD status**			
MRD^{neg} (n=464)	1 (Reference)	1 (Reference)	1 (Reference)
MRD^{pos} (n=106)	4.31 (3.14-5.93), *P*<0.001	3.36 (2.59-4.36), *P*<0.001	2.67 (2.03-3.50), *P*<0.001
**Cytogenetic risk**			
Favorable/intermediate (n=373)	1 (Reference)	1 (Reference)	1 (Reference)
Adverse (n=173)	2.27 (1.66-3.11), *P*<0.001	1.46 (1.14-1.88), *P*=0.003	1.29 (0.98-1.68), *P*=0.07
**Age at HCT (per 10 years)**	0.99 (0.98-1.01), *P*=0.37	1.01 (1.00-1.02), *P*=0.10	1.01 (1.00-1.02), *P*=0.014
**WBC at diagnosis (per 10,000/µL)**	1.00 (1.00-1.00), *P*=0.86	1.00 (1.00-1.00), *P*=0.30	1.00 (1.00-1.00), *P*=0.15
**HCT Comorbidity Index**			
0-1 (n=130)	1 (Reference)	1 (Reference)	1 (Reference)
2-3 (n=204)	0.91 (0.61-1.34), *P*=0.63	1.03 (0.75-1.41), *P*=0.87	1.08 (0.77-1.52), *P*=0.64
≥4 (n=180)	1.07 (0.72-1.59), *P*=0.72	1.29 (0.94-1.76), *P*=0.12	1.37 (0.98-1.91), *P*=0.06
**Type of AML**			
De novo (n=378)	1 (Reference)	1 (Reference)	1 (Reference)
Secondary (n=192)	1.03 (0.75-1.43), *P*=0.85	1.16 (0.91-1.49), *P*=0.24	1.20 (0.93-1.56), *P*=0.17
**Pre-HCT karyotype**			
Normalized (n=242)	1 (Reference)	1 (Reference)	1 (Reference)
Not normalized (n=92)	1.86 (1.25-2.77), *P*=0.002	1.85 (1.35-2.55), *P*<0.001	1.76 (1.25-2.46), *P*=0.001
**Pre-HCT blood counts***			
Recovered (n=425)	1 (Reference)	1 (Reference)	1 (Reference)
Not recovered (n=145)	0.66 (0.45-0.99), *P*=0.042	1.10 (0.84-1.44), *P*=0.51	1.31 (0.99-1.73), *P*=0.06
**Donor type**			
Related (n=182)	1 (Reference)	1 (Reference)	1 (Reference)
Unrelated (n=388)	0.88 (0.64-1.21), *P*=0.45	1.13 (0.87-1.46), *P*=0.36	1.19 (0.90-1.56), *P*=0.22
**HLA matching**			
Matched/identical (n=485)	1 (Reference)	1 (Reference)	1 (Reference)
9/10 matched (n=70)	1.13 (0.74-1.72), *P*=0.58	1.77 (1.30-2.42), *P*<0.001	1.84 (1.32-2.56), *P*<0.001
Haplo-identical (n=15)	2.00 (0.96-4.17), *P*=0.064	2.61 (1.42-4.80), *P*=0.002	2.58 (1.32-5.04), *P*=0.0057

*Recovered: ANC ≥1,000/µL and platelets ≥100,000/µL; not recovered: ANC <1,000/µL and/or platelets <100,000/µL. Abbreviations: ANC, absolute neutrophil count; HCT, hematopoietic cell transplantation; MAC, myeloablative conditioning; MRD, measurable residual disease; NMA, nonmyeloablative; RFS, relapse free survival; RIC, reduced-intensity conditioning; WBC, total white blood cell count.

SUPPLEMENTARY TABLE 3. Multivariable regression models for the subset of patients transplanted in first remission

	**Relapse**	**Failure for RFS**	**Overall mortality**
**Conditioning regimen**			
MAC	1 (Reference)	1 (Reference)	1 (Reference)
RIC	1.37 (0.73-2.57), *P*=0.32	1.61 (1.04-2.49), *P*=0.033	1.38 (0.86-2.21), *P*=0.18
NMA	1.95 (1.17-3.27), *P*=0.011	1.93 (1.32-2.82), *P*<0.001	1.66 (1.11-2.47), *P*=0.013
**Pre-HCT MRD status**			
MRD^{neg}	1 (Reference)	1 (Reference)	1 (Reference)
MRD^{pos}	5.43 (3.58-8.23), *P*<0.001	4.35 (3.02-6.28), *P*<0.001	3.21 (2.18-4.72), *P*<0.001
**Cytogenetic risk**			
Favorable/intermediate	1 (Reference)	1 (Reference)	1 (Reference)
Adverse	1.75 (1.14-2.69), *P*=0.011	1.24 (0.90-1.70), *P*=0.19	1.15 (0.82-1.61), *P*=0.42
**Age at HCT (per 10 years)**	0.86 (0.76-0.97), *P*=0.018	0.94 (0.85-1.05), *P*=0.28	1.01 (0.90-1.13), *P*=0.89
**WBC at diagnosis (per 10,000/μL)**	1.01 (0.99-1.04), *P*=0.37	1.03 (1.00-1.05), *P*=0.033	1.03 (1.01-1.06), *P*=0.007
**HCT Comorbidity Index**			
0-1	1 (Reference)	1 (Reference)	1 (Reference)
2-3	0.82 (0.56-1.20), *P*=0.31	0.98 (0.71-1.35), *P*=0.89	1.09 (0.77-1.54), *P*=0.62
≥4	1.04 (0.68-1.57), *P*=0.87	1.19 (0.85-1.65), *P*=0.31	1.27 (0.90-1.80), *P*=0.17
**Type of AML**			
De novo	1 (Reference)	1 (Reference)	1 (Reference)
Secondary	0.70 (0.49-1.01), *P*=0.05	0.84 (0.63-1.10), *P*=0.21	0.88 (0.66-1.18), *P*=0.40
**Pre-HCT karyotype**			
Normalized	1 (Reference)	1 (Reference)	1 (Reference)
Not normalized	1.53 (1.01-2.31), *P*=0.045	1.54 (1.09-2.18), *P*=0.014	1.41 (0.98-2.04), *P*=0.07
**Pre-HCT blood counts***			
Recovered	1 (Reference)	1 (Reference)	1 (Reference)
Not recovered	0.58 (0.23-1.79), *P*=0.40	0.92 (0.68-1.23), *P*=0.56	1.20 (0.89-1.62), *P*=0.24
**Interaction RIC-MRD^{pos}**	0.64 (0.23-1.79), *P*=0.40	0.57 (0.26-1.24), *P*=0.16	0.82 (0.37-1.84), *P*=0.63
**Interaction NMA-MRD^{pos}**	0.62 (0.29-1.34), *P*=0.23	0.55 (0.29-1.04), *P*=0.07	0.58 (0.30-1.13), *P*=0.11

*Recovered: ANC ≥1,000/μL and platelets ≥100,000/μL; not recovered: ANC <1,000/μL and/or platelets <100,000/μL Abbreviations: ANC, absolute neutrophil count; HCT, hematopoietic cell transplantation; MAC, myeloablative conditioning; MRD, measurable residual disease; NMA, nonmyeloablative; RFS, relapse free survival; RIC, reduced-intensity conditioning; WBC, total white blood cell count.

SUPPLEMENTARY TABLE 4. Demographic and clinical characteristics of the subset of patients who underwent fully HLA-matched related- or unrelated-donor HCT, stratified by conditioning intensity

	**MAC (n=381)**	**RIC (n=103)**	**NMA (n=142)**	**All patients (n=626)**	***P*-value**
**Median age at diagnosis (range), years**	49 (18-71)	62 (20-74)	65 (19-77)	54 (18-77)	<0.001
**Median age at HCT (range), years**	50 (18-73)	63 (23-75)	66 (20-80)	55 (18-80)	<0.001
**Male gender, n (%)**	191 (50)	51 (50)	83 (58)	326 (52)	0.22
**Median WBC at diagnosis (range), x10^3/µL**	11 (0-297)	5 (0-348)	3 (1-295)	8 (0-348)	0.001
**Cytogenetics, n (%)**					0.51
Favorable	29 (9)	3 (3)	6 (4)	38 (6)	
Intermediate	241 (63)	70 (68)	89 (63)	400 (64)	
Adverse	93 (24)	27 (26)	39 (27)	159 (25)	
Missing	18 (5)	3 (3)	8 (6)	29 (5)	
**Remission status, n (%)**					0.039
First remission	286 (75)	78 (76)	121 (85)	485 (77)	
Second remission	95 (25)	25 (24)	21 (15)	141 (23)	
**Pre-HCT MRD status, n (%)**					0.67
MRD^{neg}	306 (80)	85 (83)	119 (84)	510 (81)	
MRD^{pos}	75 (20)	18 (17)	23 (16)	116 (19)	
Median % abnormal blasts (range)	0.48 (0.007-19.4)	0.655 (0.007-5)	0.2 (0.01-2.7)	0.4 (0.007-19.4)	0.07
**Secondary AML, n (%)**	82 (22)	40 (39)	55 (39)	177 (28)	<0.001
**Median CR duration before HCT (range), days**	92 (7-485)	81 (11-455)	107 (16-356)	94 (7-485)	0.009
**Recovered peripheral blood counts before HCT*, n (%)**	281 (74)	71 (69)	94 (66)	446 (71)	0.20
**Recovered ANC before HCT*, n (%)**	356 (93)	92 (89)	132 (93)	580 (93)	0.35
**Recovered platelet count before HCT*, n (%)**	282 (74)	72 (70)	95 (67)	449 (72)	0.25
**Routine cytogenetics before HCT, n (%)**					0.40
Normalized karyotype	150 (39)	37 (36)	54 (38)	241 (39)	
Abnormal karyotype	65 (17)	17 (17)	16 (11)	98 (16)	
Non-informative karyotype**	154 (40)	46 (45)	65 (46)	265 (42)	
Missing	12 (3)	3 (3)	7 (5)	22 (4)	
**HCT Comorbidity Index, n (%)**					<0.001
0-1	99 (26)	11 (11)	31 (22)	141 (23)	
2-3	139 (36)	40 (39)	44 (31)	223 (36)	
≥4	94 (25)	43 (42)	61 (43)	198 (32)	
Missing	49 (13)	9 (9)	6 (4)	181 (30)	
**Unrelated donor, n (%)**	230 (60)	78 (76)	104 (73)	412 (66)	0.002

**Patient / donor CMV status**					0.82
Neg / neg	95 (26)	20 (20)	39 (28)	154 (25)	
Neg / pos	45 (12)	14 (14)	19 (13)	78 (13)	
Pos / neg	118 (32)	36 (37)	44 (31)	198 (32)	
Pos / pos	114 (31)	28 (29)	39 (28)	181 (30)	
**Conditioning regimen, n (%)**					<0.001
MAC					
Containing high-dose TBI (≥12 Gy)	55 (14)	0	0	55 (9)	
Not containing high-dose TBI	326 (86)	0	0	326 (52)	
RIC	0	103 (100%)	0	142 (23)	
NMA	0	0	142 (100)	103 (16)	
**Source of stem cells, n (%)**					<0.001
PBSC	331 (87)	101 (98)	142 (100)	574 (92)	
BM	50 (13)	2 (2)	0	52 (8)	
**GVHD prophylaxis, n (%)**					<0.001
CNI + MMF ± sirolimus	57 (15)	74 (72)	137 (96)	268 (43)	
CNI + MTX ± other	261 (69)	22 (21)	0	283 (45)	
PTCy	50 (13)	7 (7)	4 (3)	61 (10)	
Other	13 (3)	0	1 (1)	14 (2)	

*ANC ≥1,000/μL and platelets ≥100,000/μL; **normal cytogenetics in patient with cytogenetically normal AML or missing cytogenetics at diagnosis. Abbreviations: ANC, absolute neutrophil count; BM, bone marrow; CNI, calcineurin inhibitor; HCT, hematopoietic cell transplantation; MAC, myeloablative conditioning; MMF, mycophenolate mofetil; MTX, methotrexate; NMA, nonmyeloablative; PBSC, peripheral blood stem cells; PTCy, post transplantation cyclophosphamide; RIC, reduced intensity conditioning; TBI, total body irradiation; WBC, total white blood cell count.

SUPPLEMENTARY TABLE 5. Univariate regression models for the subset of patients who underwent fully HLA-matched related- or unrelated-donor HCT

	Relapse	Failure for RFS	Overall mortality
**Conditioning regimen**			
MAC (n=381)	1 (Reference)	1 (Reference)	1 (Reference)
RIC (n=103)	1.05 (0.69-1.59), *P*=0.82	1.40 (1.02-1.93), *P*=0.04	1.43 (1.02-2.02), *P*=0.041
NMA (n=142)	1.45 (1.04-2.03), *P*=0.029	1.62 (1.25-2.11), *P*<0.001	1.55 (1.18-2.04), *P*=0.002
**Pre-HCT MRD status**			
MRD^{neg} (n=510)	1 (Reference)	1 (Reference)	1 (Reference)
MRD^{pos} (n=116)	4.60 (3.43-6.19), *P*<0.001	3.36 (2.62-4.31), *P*<0.001	2.50 (1.92-3.24), *P*<0.001
**Remission status**			
First remission (n=485)	1 (Reference)	1 (Reference)	1 (Reference)
Second remission (n=141)	1.51 (1.09-2.08), P=0.012	1.57 (1.22-2.02), *P*<0.001	1.55 (1.19-2.02), *P*=0.001
**Cytogenetic risk**			
Favorable/intermediate (n=438)	1 (Reference)	1 (Reference)	1 (Reference)
Adverse (n=159)	2.15 (1.60-2.89), *P*<0.001	1.35 (1.05-1.74), *P*=0.020	1.19 (0.90-1.56), *P*=0.22
**Age at HCT (per 10 years)**	1.01 (1.00-1.02), *P*=0.27	1.01 (1.00-1.02), *P*=0.003	1.02 (1.00-1.00), *P*<0.001
**WBC at diagnosis (per 10,000/µL)**	1.00 (1.00-1.00), *P*=0.55	1.00 (1.00-1.00), *P*=0.24	1.00 (1.00-1.04), *P*=0.89
**HCT Comorbidity Index**			
0-1 (n=141)	1 (Reference)	1 (Reference)	1 (Reference)
2-3 (n=223)	0.99 (0.68-1.44), *P*=0.95	1.09 (0.80-1.47), *P*=0.59	1.11 (0.81-1.53), *P*=0.51
≥4 (n=198)	1.00 (0.68-1.46), *P*=0.98	1.22 (0.90-1.66), *P*=0.20	1.29 (0.93-1.77), *P*=0.13
**Type of AML**			
De novo (n=449)	1 (Reference)	1 (Reference)	1 (Reference)
Secondary (n=177)	1.01 (0.73-1.39), *P*=0.96	1.07 (0.83-1.37), *P*=0.61	1.10 (0.85-1.42), *P*=0.49
**Pre-HCT karyotype**			
Normalized (n=240)	1 (Reference)	1 (Reference)	1 (Reference)
Not normalized (n=98)	2.36 (1.61-3.45), *P*<0.001	2.31 (1.69-3.17), *P*<0.001	2.17 (1.55-3.03), *P*<0.001
**Pre-HCT blood counts***			
Recovered (n=446)	1 (Reference)	1 (Reference)	1 (Reference)
Not recovered (n=180)	0.90 (0.65-1.25), *P*=0.53	1.34 (1.05-1.72), *P*=0.018	1.51 (1.17-1.95), *P*=0.001
**Donor type**			
Related (n=214)	1 (Reference)	1 (Reference)	1 (Reference)
Unrelated (n=412)	0.98 (0.73-1.31), *P*=0.88	1.10 (0.86-1.40), *P*=0.45	1.14 (0.89-1.47), *P*=0.30

*Recovered: ANC ≥1,000/μL and platelets ≥100,000/μL; not recovered: ANC <1,000/μL and/or platelets <100,000/μL. Abbreviations: ANC, absolute neutrophil count; HCT, hematopoietic cell transplantation; MAC, myeloablative conditioning; MRD, measurable residual disease; NRM, non-relapse mortality; RFS, relapse free survival; RIC, reduced-intensity conditioning; WBC, total white blood cell count.

SUPPLEMENTARY TABLE 6. Multivariable regression models for the subset of patients who underwent fully HLA-matched related- or unrelated-donor HCT

	Relapse	Failure for RFS	Overall mortality
**Conditioning regimen**			
MAC	1 (Reference)	1 (Reference)	1 (Reference)
RIC	1.52 (0.85-2.69), *P*=0.16	1.80 (1.19-2.73), *P*=0.005	1.65 (1.07-2.57), *P*=0.025
NMA	2.83 (1.74-4.61), *P*<0.002	2.27 (1.57-3.28), *P*<0.001	1.82 (1.24-2.66), *P*=0.002
**Pre-HCT MRD status**			
MRD^{neg}	1 (Reference)	1 (Reference)	1 (Reference)
MRD^{pos}	6.08 (4.10-9.04), *P*<0.001	4.01 (2.85-5.66), *P*<0.001	2.77 (1.94-3.97), *P*<0.001
**Remission status**			
First remission	1 (Reference)	1 (Reference)	1 (Reference)
Second remission	1.93 (1.33-2.82), *P*<0.001	1.67 (1.25-2.23), *P*=0.001	1.43 (1.06-1.94), *P*=0.019
**Cytogenetic risk**			
Favorable/intermediate	1 (Reference)	1 (Reference)	1 (Reference)
Adverse	1.96 (1.30-2.96), *P*=0.001	1.27 (0.92-1.74), *P*=0.15	1.24 (0.89-1.73), *P*=0.21
**Age at HCT (per 10 years)**	0.96 (0.85-1.09), *P*=0.54	1.01 (0.92-1.12), *P*=0.79	1.05 (0.94-1.17), *P*=0.42
**WBC at diagnosis (per 10,000/µL)**	1.00 (0.98-1.03), *P*=0.87	1.02 (1.00-1.04), *P*=0.05	1.02 (1.00-1.04), *P*=0.039
**HCT Comorbidity Index**			
0-1	1 (Reference)	1 (Reference)	1 (Reference)
2-3	1.00 (0.68-1.46), *P*=0.99	1.07 (0.79-1.47), *P*=0.65	1.09 (0.78-1.51), *P*=0.62
≥4	0.96 (0.64-1.44), *P*=0.84	1.11 (0.80-1.54), *P*=0.52	1.18 (0.85-1.65), *P*=0.32
**Type of AML**			
De novo	1 (Reference)	1 (Reference)	1 (Reference)
Secondary	0.71 (0.49-1.01), *P*=0.06	0.78 (0.58-1.04), *P*=0.085	0.88 (0.65-1.18), *P*=0.39
**Pre-HCT karyotype**			
Normalized	1 (Reference)	1 (Reference)	1 (Reference)
Not normalized	1.87 (1.25-2.79), *P*=0.002	1.81 (1.28-2.57), *P*<0.001	1.68 (1.16-2.43), *P*=0.006
**Pre-HCT blood counts***			
Recovered	1 (Reference)	1 (Reference)	1 (Reference)
Not recovered	0.71 (0.49-1.02), *P*=0.06	1.04 (0.80-1.36), *P*=0.75	1.29 (0.98-1.69), *P*=0.07
**Interaction RIC-MRD^{pos}**	0.66 (0.27-1.61), *P*=0.37	0.60 (0.29-1.28), *P*=0.19	0.46 (0.20-1.05), *P*=0.06
**Interaction NMA-MRD^{pos}**	0.33 (0.16-0.70), *P*=0.004	0.47 (0.25-0.89), *P*=0.02	0.53 (0.28-1.03), *P*=0.06

*Recovered: ANC ≥1,000/µL and platelets ≥100,000/µL; not recovered: ANC <1,000/µL and/or platelets <100,000/µL. Abbreviations: ANC, absolute neutrophil count; HCT, hematopoietic cell transplantation; MAC, myeloablative conditioning; MRD, measurable residual disease; NMA, nonmyeloablative; RFS, relapse free survival; RIC, reduced-intensity conditioning; WBC, total white blood cell count.

SUPPLEMENTARY TABLE 7. Pre-transplantation demographic and clinical characteristics for the subset of patients who received MAC and RIC regimens similar to those used in the BMT CTN 0901 trial, stratified by conditioning intensity

	MAC (n=215)	RIC (n=65)	All patients (n=280)	*P*-value
**Median age at diagnosis (range), years**	49 (18-65)	64 (20-74)	52 (18-74)	<0.001
**Median age at HCT (range), years**	49 (18-66)	64 (23-75)	53 (18-75)	<0.001
**Male gender, n (%)**	124 (58)	33 (51)	157 (56)	0.39
**Median WBC at diagnosis (range), x10^3/µL**	13 (0-297)	9.5 (0-348)	11 (0-348)	0.42
**Cytogenetics, n (%)**				0.47
Favorable	16 (7)	3 (5)	19 (7)	
Intermediate	135 (63)	48 (74)	183 (65)	
Adverse	57 (27)	12 (18)	69 (25)	
Missing	7 (3)	2 (3)	9 (3)	
**Remission status, n (%)**				0.50
First remission	169 (79)	48 (74)	217 (78)	
Second remission	46 (21)	17(26)	63 (22)	
**Pre-HCT MRD status, n (%)**				0.49
MRD^{neg}	169 (79)	54 (83)	223 (80)	
MRD^{pos}	46 (21)	11 (17)	57 (20)	
**Median % abnormal blasts (range)**	0.95 (0.007-8)	0.70 (0.07-5)	0.70 (0.007-8)	0.99
**Secondary AML, n (%)**	46 (21)	22 (34)	68 (24)	0.048
**Median CR duration before HCT (range), days**	99.5 (11-485)	87 (11-455)	94 (11-485)	0.68
**Recovered peripheral blood counts before HCT*, n (%)**	171 (80)	42 (65)	213 (76)	0.02
**Recovered ANC before HCT*, n (%)**	203 (94)	57 (88)	260 (93)	0.095
**Recovered platelet count before HCT*, n (%)**	172 (80)	43 (66)	215 (77)	0.029
**Routine cytogenetics before HCT, n (%)**				0.14
Normalized karyotype	93 (43)	21 (32)	114 (41)	
Abnormal karyotype	33 (15)	8 (12)	41 (15)	
Non-informative karyotype**	84 (39)	32 (49)	116 (41)	
Missing	5 (2)	4 (6)	9 (3)	
**HCT Comorbidity Index, n (%)**				0.0095
0-1	71 (33)	9 (14)	80 (29)	
2-3	105 (49)	39 (60)	144 (51)	
4	39 (18)	17 (26)	56 (20)	

**Unrelated donor, n (%)**	150 (70)	55 (85)	205 (73)	0.017
**Patient / donor CMV status**				0.19
Neg / neg	63 (30)	12 (19)	75 (28)	
Neg / pos	29 (14)	13 (21)	42 (15)	
Pos / neg	71 (34)	19 (31)	90 (33)	
Pos / pos	47 (22)	18 (29)	65 (24)	
**Conditioning regimen, n (%)**				<0.001
MAC				
Containing high-dose TBI (≥12 Gy)	28 (13)	0 (0)	28 (10)	
Not containing high-dose TBI	187 (87)	0 (0)	187 (67)	
RIC	0 (0)	65 (100)	65 (23)	
**Source of stem cells, n (%)**				<0.001
PBSC	168 (78)	62 (95)	230 (82)	
BM	47 (22)	3 (5)	50 (18)	
**GVHD prophylaxis, n (%)**				<0.001
CNI + MMF ± sirolimus	0 (0)	43 (66)	43 (15)	
CNI + MTX ± other	177 (82)	16 (25)	193 (69)	
PTCy	38 (18)	6 (9)	44 (16)	

*ANC ≥1,000/μL and platelets ≥100,000/μL; **normal cytogenetics in patient with cytogenetically normal AML or missing cytogenetics at diagnosis. Abbreviations: ANC, absolute neutrophil count; BM, bone marrow; CNI, calcineurin inhibitor; HCT, hematopoietic cell transplantation; MAC, myeloablative conditioning; MMF, mycophenolate mofetil; MTX, methotrexate; PBSC, peripheral blood stem cells; PTCy, post transplantation cyclophosphamide; RIC, reduced intensity conditioning; TBI, total body irradiation; WBC, total white blood cell count.

SUPPLEMENTARY TABLE 8. Univariate regression models for the subset of patients who received MAC and RIC regimens similar to those used in the BMT CTN 0901 trial

	Relapse	Failure for RFS	Overall mortality
**Conditioning regimen**			
MAC (n=215)	1 (Reference)	1 (Reference)	1 (Reference)
RIC (n=65)	0.83 (0.47-1.44), *P*=0.50	1.54 (1.05-2.27), *P*=0.028	1.58 (1.05-2.39), *P*=0.03
**Pre-HCT MRD status**			
MRD^{neg} (n=223)	1 (Reference)	1 (Reference)	1 (Reference)
MRD^{pos} (n=57)	5.54 (3.60-8.52), *P*<0.001	3.91 (2.73-5.61), *P*<0.001	3.12 (2.15-4.53), *P*<0.001
**Remission status**			
First remission (n=217)	1 (Reference)	1 (Reference)	1 (Reference)
Second remission (n=63)	1.25 (0.76-2.05), *P*=0.38	1.34 (0.92-1.96), *P*=0.13	1.27 (0.85-1.91), *P*=0.24
**Cytogenetic risk**			
Favorable/intermediate (n=202)	1 (Reference)	1 (Reference)	1 (Reference)
Adverse (n=69)	2.26 (1.46-3.51), *P*<0.001	1.33 (0.91-1.93), *P*=0.14	1.14 (0.76-1.71), *P*=0.51
**Age at HCT (per 10 years)**	0.99 (0.98-1.01), *P*=0.30	1.01 (1.00-1.02), *P*=0.21	1.01 (1.00-1.02), *P*=0.18
**WBC at diagnosis (per 10,000/µL)**	1.00 (1.00-1.00), *P*=0.34	1.00 (1.00-1.00), *P*=0.14	1.00 (1.00-1.00), *P*=0.27
**HCT Comorbidity Index**			
0-1 (n=80)	1 (Reference)	1 (Reference)	1 (Reference)
2-3 (n=144)	1.02 (0.64-1.62), *P*=0.94	1.10 (0.75-1.61), *P*=0.61	1.08 (0.72-1.62), *P*=0.71
4 (n=56)	0.85 (0.46-1.57), *P*=0.61	0.87 (0.52-1.44), *P*=0.58	0.88 (0.51-1.50), *P*=0.63
**Type of AML**			
De novo (n=212)	1 (Reference)	1 (Reference)	1 (Reference)
Secondary (n=68)	0.88 (0.53-1.46), *P*=0.62	1.06 (0.72-1.57), *P*=0.76	1.12 (0.75-1.69), *P*=0.57
**Pre-HCT karyotype**			
Normalized (n=113)	1 (Reference)	1 (Reference)	1 (Reference)
Not normalized (n=41)	2.28 (1.32-3.95), *P*=0.0032	1.89 (1.17-3.04), *P*=0.0089	1.99 (1.20-3.29), *P*=0.0077
**Pre-HCT blood counts***			
Recovered (n=213)	1 (Reference)	1 (Reference)	1 (Reference)
Not recovered (n=67)	1.15 (0.71-1.86), *P*=0.58	1.45 (0.99-2.11), *P*=0.053	1.39 (0.93-2.08), *P*=0.10
**Donor type**			
Related (n=75)	1 (Reference)	1 (Reference)	1 (Reference)
Unrelated (n=205)	1.13 (0.70-1.83), *P*=0.61	1.37 (0.93-2.03), *P*=0.11	1.43 (0.94-2.18), *P*=0.091
**HLA matching**			
Matched/identical (n=236)	1 (Reference)	1 (Reference)	1 (Reference)

9/10 matched (n=44)	1.51 (0.95-2.40), *P*=0.082	1.98 (1.34-2.93), *P*<0.001	1.80 (1.18-2.75), *P*=0.0065

*Recovered: ANC ≥1,000/µL and platelets ≥100,000/µL; not recovered: ANC <1,000/µL and/or platelets <100,000/µL. Abbreviations: ANC, absolute neutrophil count; HCT, hematopoietic cell transplantation; MAC, myeloablative conditioning; MRD, measurable residual disease; RFS, relapse free survival; RIC, reduced-intensity conditioning; WBC, total white blood cell count.

SUPPLEMENTARY TABLE 9. Multivariable regression models for the subset of patients who received MAC and RIC regimens similar to those used in the BMT CTN 0901 trial

	**Relapse**	**Failure for RFS**	**Overall mortality**
**Conditioning regimen**			
MAC	1 (Reference)	1 (Reference)	1 (Reference)
RIC	1.02 (0.41-2.54), *P*=0.97	1.98 (1.17-3.35), *P*=0.011	2.22 (1.28-3.87), *P*=0.0046
**Pre-HCT MRD status**			
MRD^{neg}	1 (Reference)	1 (Reference)	1 (Reference)
MRD^{pos}	6.82 (4.10-11.34), *P*<0.001	5.32 (3.38-8.39), *P*<0.001	4.57 (2.83-7.37), *P*<0.001
**Remission status**			
First remission	1 (Reference)	1 (Reference)	1 (Reference)
Second remission	0.92 (0.53-1.60), *P*=0.76	1.17 (0.77-1.78), *P*=0.46	1.11 (0.71-1.74), *P*=0.65
**Cytogenetic risk**			
Favorable/intermediate	1 (Reference)	1 (Reference)	1 (Reference)
Adverse	2.90 (1.52-5.52), *P*=0.0012	1.67 (1.04-2.68), *P*=0.034	1.25 (0.76-2.07), *P*=0.38
**Age at HCT (per 10 years)**	0.83 (0.70-0.99), *P*=0.037	0.96 (0.83-1.10), *P*=0.56	0.98 (0.84-1.14), *P*=0.75
**WBC at diagnosis (per 10,000/μL)**	1.02 (0.99-1.06), *P*=0.14	1.02 (0.99-1.05), *P*=0.15	1.01 (0.98-1.04), *P*=0.68
**HCT Comorbidity Index**			
0-1	1 (Reference)	1 (Reference)	1 (Reference)
2-3	0.91 (0.56-1.48), *P*=0.71	1.00 (0.67-1.49), *P*=0.99	0.95 (0.62-1.45), *P*=0.82
4	1.01 (0.52-1.97), *P*=0.98	0.85 (0.50-1.48), *P*=0.57	0.81 (0.46-1.44), *P*=0.48
**Type of AML**			
De novo	1 (Reference)	1 (Reference)	1 (Reference)
Secondary	0.43 (0.23-0.81), *P*=0.009	0.64 (0.41-1.02), *P*=0.061	0.71 (0.44-1.15), *P*=0.17
**Pre-HCT karyotype**			
Normalized	1 (Reference)	1 (Reference)	1 (Reference)
Not normalized	1.89 (1.04-3.43), *P*=0.036	1.56 (0.93-2.61), *P*=0.092	1.59 (0.92-2.74), *P*=0.094
**Pre-HCT blood counts***			
Recovered	1 (Reference)	1 (Reference)	1 (Reference)
Not recovered	1.63 (0.94-2.83), *P*=0.084	1.39 (0.92-2.11), *P*=0.12	1.33 (0.85-2.07), *P*=0.22
**Interaction RIC - MRD^{pos}**	2.45 (0.65-9.26), *P*=0.19	0.69 (0.27-1.80), *P*=0.45	0.28 (0.09-0.85), *P*=0.025

*Recovered: ANC ≥1,000/μL and platelets ≥100,000/μL; not recovered: ANC <1,000/μL and/or platelets <100,000/μL. Abbreviations: ANC, absolute neutrophil count; HCT, hematopoietic cell transplantation; MAC, myeloablative conditioning; MRD, measurable residual disease; RFS, relapse free survival; RIC, reduced-intensity conditioning; WBC, total white blood cell count.

SUPPLEMENTARY FIGURE 1

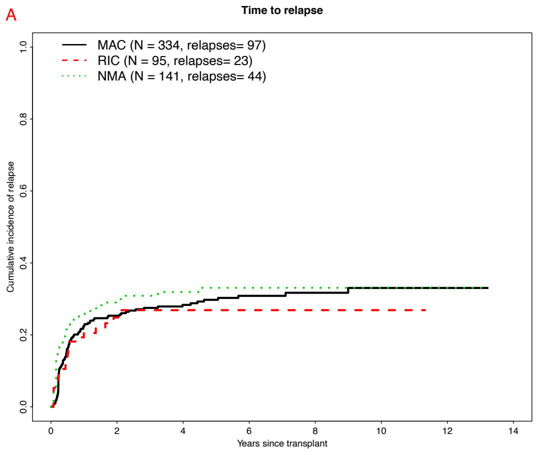

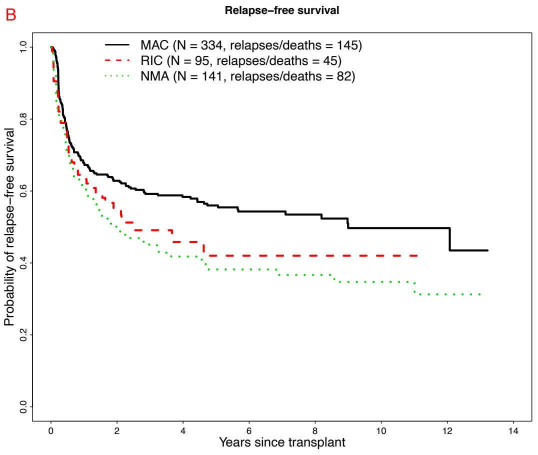

334	179	132	87	54	25	10	MAC
95	33	12	8	2	2		RIC
141	55	37	25	20	16	6	NMA

334	179	132	87	54	25	10	MAC
95	33	12	8	2	2		RIC
141	55	37	25	20	16	6	NMA

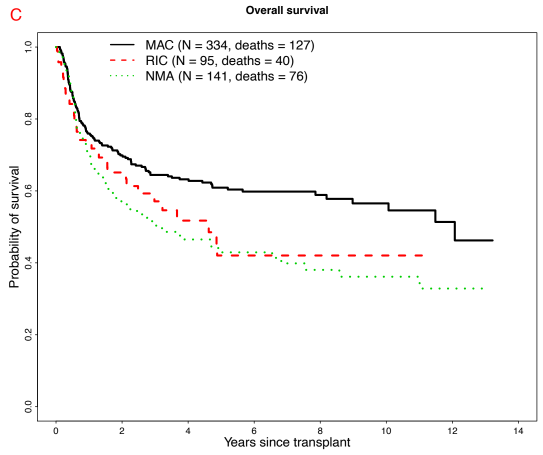

334	200	145	98	60	30	12	MAC
95	39	16	10	2	2		RIC
141	63	43	28	21	17	7	NMA

Post-transplant outcome for 570 adults with AML undergoing allogeneic HCT while in first morphologic remission, stratified by conditioning intensity. Estimates of **(A)** cumulative risk of relapse, **(B)** relapse-free survival, and **(C)** overall survival following allogeneic HCT. Outcome estimates are shown individually for MAC patients (n=334), RIC patients (n=95), and NMA HCT patients (n=141), respectively.

SUPPLEMENTARY FIGURE 2

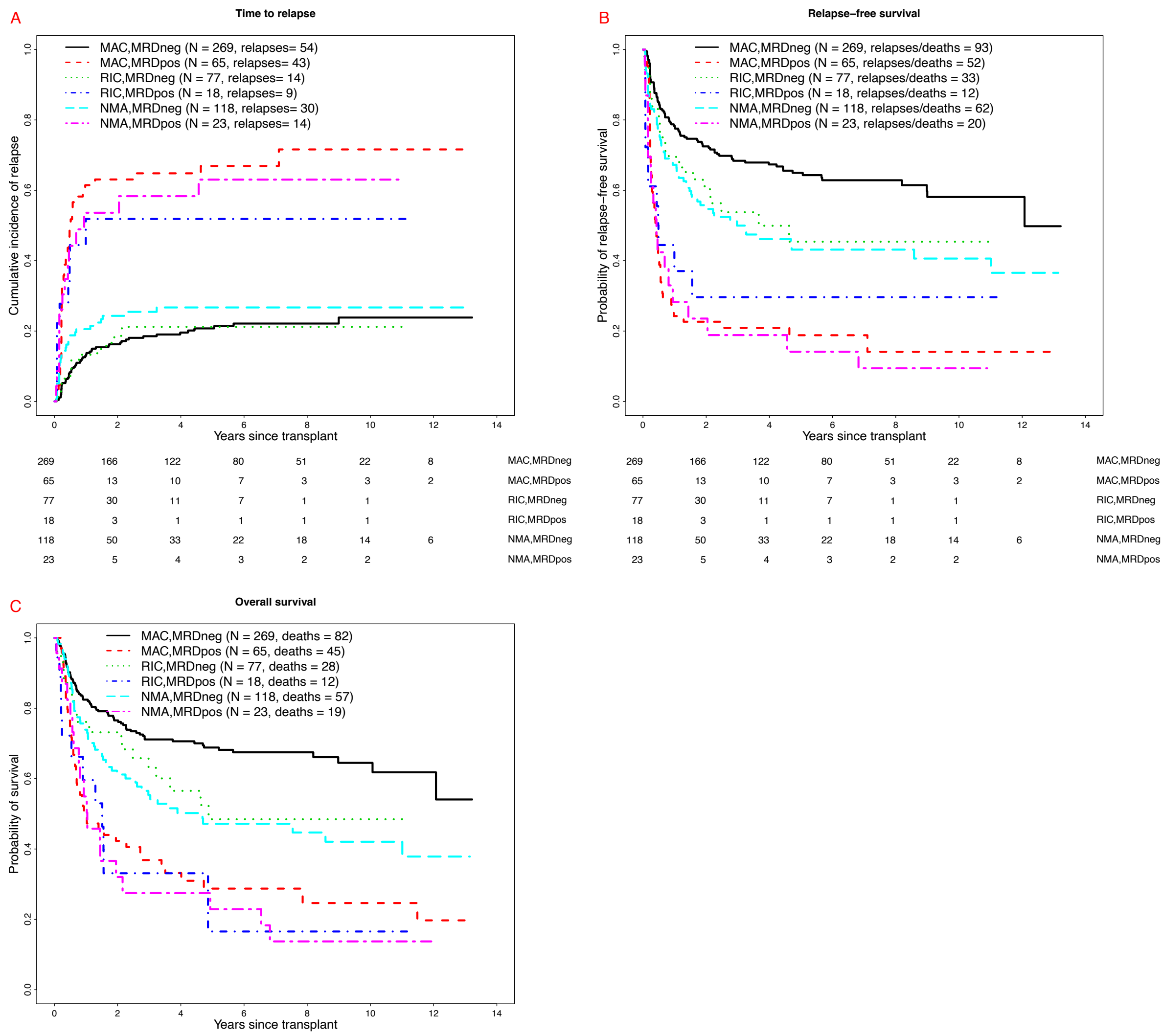

A

269	166	122	80	51	22	8	MAC,MRDneg
65	13	10	7	3	3	2	MAC,MRDpos
77	30	11	7	1	1		RIC,MRDneg
18	3	1	1	1	1		RIC,MRDpos
118	50	33	22	18	14	6	NMA,MRDneg
23	5	4	3	2	2		NMA,MRDpos

B

269	166	122	80	51	22	8	MAC,MRDneg
65	13	10	7	3	3	2	MAC,MRDpos
77	30	11	7	1	1		RIC,MRDneg
18	3	1	1	1	1		RIC,MRDpos
118	50	33	22	18	14	6	NMA,MRDneg
23	5	4	3	2	2		NMA,MRDpos

C

269	176	130	88	55	25	9	MAC,MRDneg
65	24	15	10	5	5	3	MAC,MRDpos
77	35	14	9	1	1		RIC,MRDneg
18	4	2	1	1	1		RIC,MRDpos
118	56	37	23	18	14	6	NMA,MRDneg
23	7	6	5	3	3	1	NMA,MRDpos

Post-transplant outcome for 570 adults with AML undergoing allogeneic HCT while in first morphologic remission, stratified by conditioning intensity and pre-transplant MRD status. Estimates of **(A)** cumulative risk of relapse, **(B)** relapse-free survival, and **(C)** overall survival following allogeneic HCT. Outcome estimates are shown individually for MAC patients in MRD^{neg} remission (n=269) or MRD^{pos} remission (n=65), RIC patients in MRD^{neg} remission (n=77) or MRD^{pos} remission (n=18), and NMA HCT patients in MRD^{neg} remission (n=118) or MRD^{pos} remission (n=23), respectively.

SUPPLEMENTARY FIGURE 3

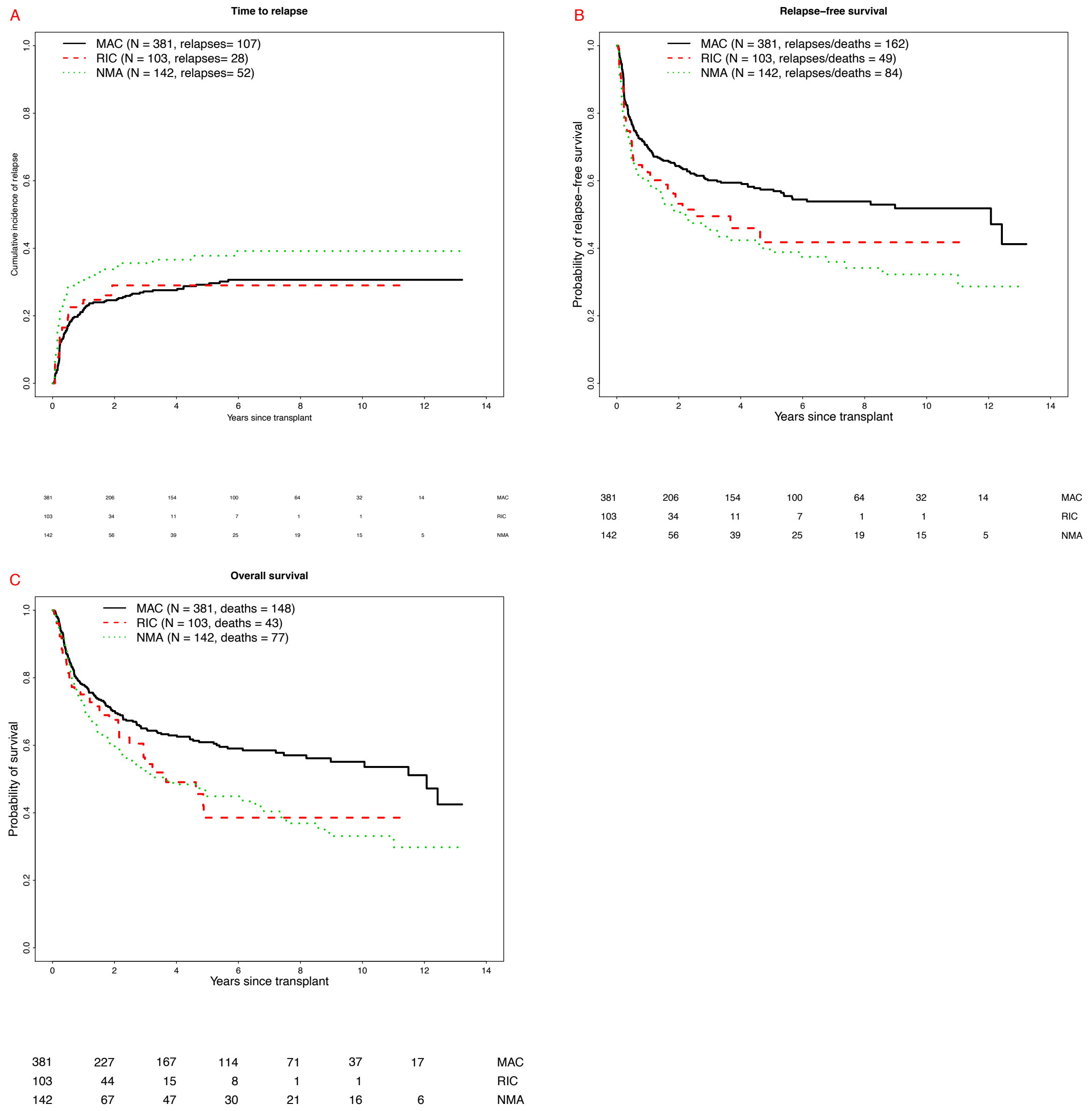

Post-transplant outcome for 626 adults with AML undergoing HCT with an HLA-identical/matched allograft, stratified by conditioning intensity. Estimates of **(A)** cumulative risk of relapse, **(B)** relapse-free survival, and **(C)** overall survival following allogeneic HCT. Outcome estimates are shown individually for MAC patients (n=381), RIC patients (n=103), and NMA HCT patients (n=142), respectively.

SUPPLEMENTARY FIGURE 4

A

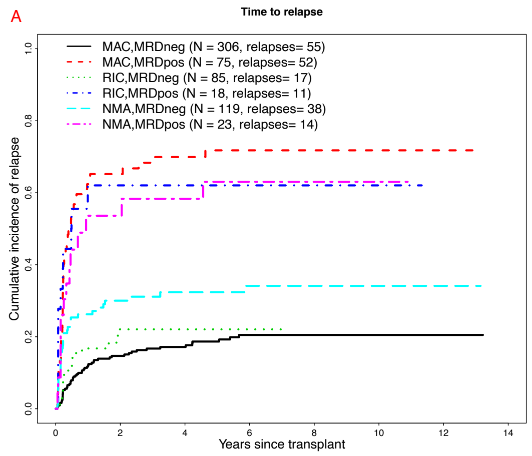

306	190	143	92	60	29	11	MAC,MRDneg
75	16	11	8	4	3	3	MAC,MRDpos
85	31	10	6				RIC,MRDneg
18	3	1	1	1	1		RIC,MRDpos
119	50	35	22	17	13	5	NMA,MRDneg
23	6	4	3	2	2		NMA,MRDpos

B

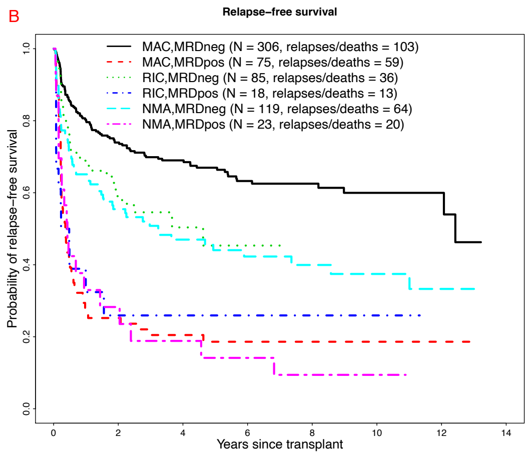

306	190	143	92	60	29	11	MAC,MRDneg
75	16	11	8	4	3	3	MAC,MRDpos
85	31	10	6				RIC,MRDneg
18	3	1	1	1	1		RIC,MRDpos
119	50	35	22	17	13	5	NMA,MRDneg
23	6	4	3	2	2		NMA,MRDpos

C

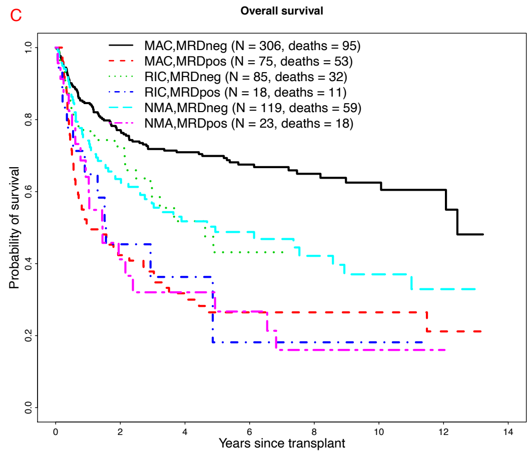

306	199	149	102	64	32	13	MAC,MRDneg
75	28	18	12	7	5	4	MAC,MRDpos
85	38	12	7				RIC,MRDneg
18	6	3	1	1	1		RIC,MRDpos
119	58	40	25	18	13	5	NMA,MRDneg
23	9	7	5	3	3	1	NMA,MRDpos

Post-transplant outcome for 626 adults with AML undergoing HCT with an HLA-identical/matched allograft, stratified by conditioning intensity and pre-transplant MRD status. Estimates of **(A)** cumulative risk of relapse, **(B)** relapse-free survival, and **(C)** overall survival following allogeneic HCT. Outcome estimates are shown individually for MAC patients in MRD^{neg} remission (n=306) or MRD^{pos} remission (n=75), RIC patients in MRD^{neg} remission (n=85) or MRD^{pos} remission (n=18), and NMA HCT patients in MRD^{neg} remission (n=119) or MRD^{pos} remission (n=23), respectively.

SUPPLEMENTARY FIGURE 5

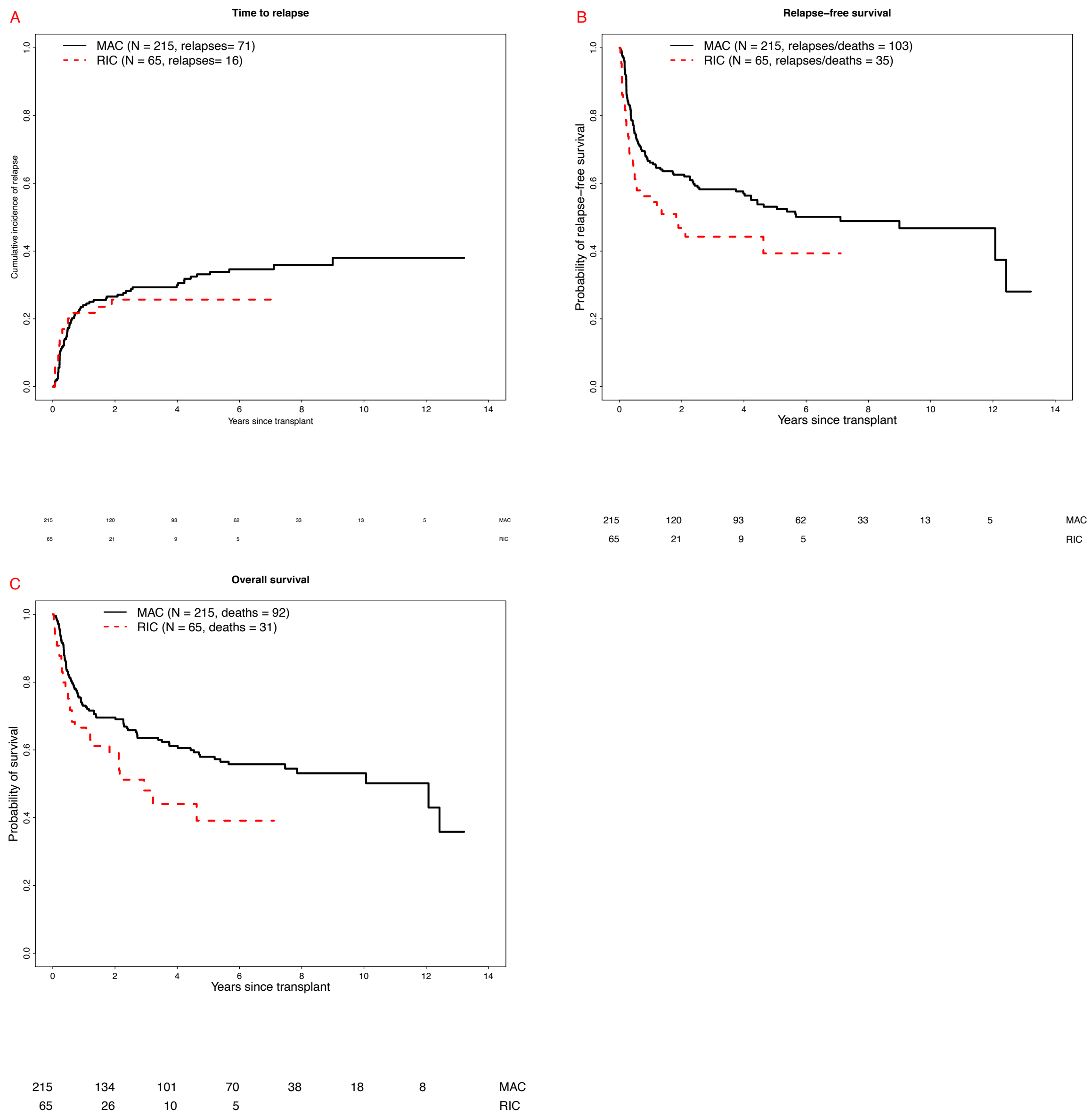

Post-transplant outcome for 280 adults with AML undergoing allogeneic HCT while in first or second morphologic remission after receiving MAC or RIC regimens similar to those used in the BMT CTN 0901 trial, stratified by conditioning intensity. Estimates of **(A)** cumulative risk of relapse, **(B)** relapse-free survival, and **(C)** overall survival following allogeneic HCT. Outcome estimates are shown individually for MAC patients (n=215) and RIC patients (n=65), respectively.

SUPPLEMENTARY FIGURE 6

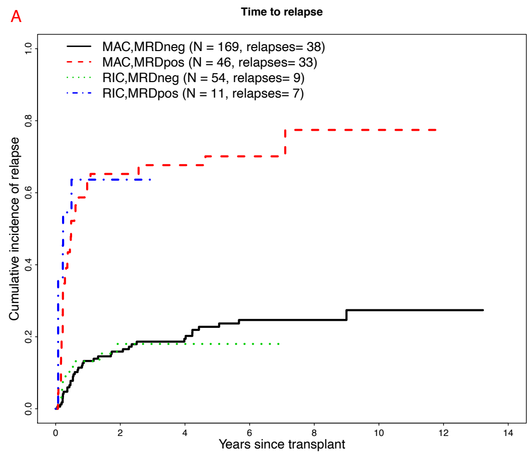

169	112	86	58	32	12	5	MAC,MRDneg
46	8	7	4	1	1		MAC,MRDpos
54	19	9	5				RIC,MRDneg
11	2						RIC,MRDpos

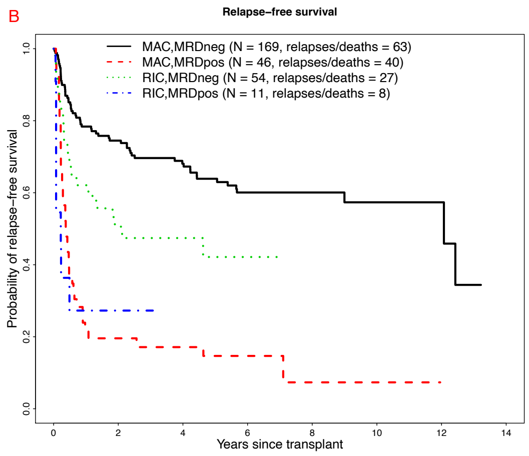

169	112	86	58	32	12	5	MAC,MRDneg
46	8	7	4	1	1		MAC,MRDpos
54	19	9	5				RIC,MRDneg
11	2						RIC,MRDpos

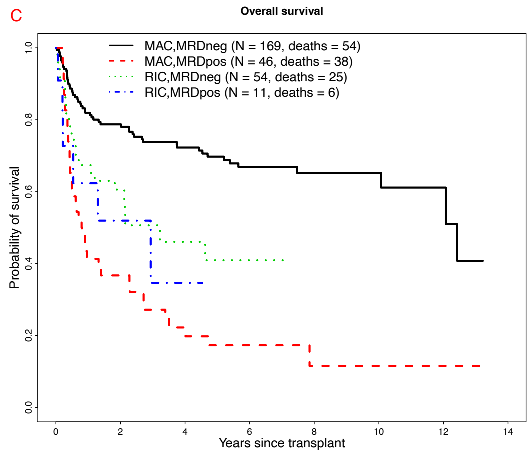

169	118	92	65	36	16	7	MAC,MRDneg
46	16	9	5	2	2	1	MAC,MRDpos
54	22	9	5				RIC,MRDneg
11	4	1					RIC,MRDpos

Post-transplant outcomes for 280 adults with AML undergoing allogeneic HCT while in first or second morphologic remission after receiving MAC or RIC regimens similar to those used in the BMT CTN 0901 trial, stratified by conditioning intensity and pre-transplant MRD status. Estimates of **(A)** cumulative risk of relapse, **(B)** relapse-free survival, and **(C)** overall survival following allogeneic HCT. Outcome estimates are shown individually for MAC patients in MRD^{neg} remission (n=169) or MRD^{pos} remission (n=46) as well as RIC patients in MRD^{neg} remission (n=54) or MRD^{pos} remission (n=11), respectively.
